# Smart Egg Processing in the Digital Food Era, Part II: AI-Optimized Non-Thermal Processing, Functional Preservation, and Industrial Implementation

**DOI:** 10.3390/foods15213952

**Published:** 2026-11-03

**Authors:** Osama K. Abou-Emera, Mohammed Aladhadh

**Affiliations:** 1 Department of Animal and Poultry Production, College of Agriculture and Food, Qassim University, Buraydah 51452, Saudi Arabia; 2 Department of Poultry Breeding Research, Animal Production Research Institute, Agriculture Research Centre, Dokki, Giza 12618, Egypt; 3 Department of Food Science and Human Nutrition, College of Agriculture and Food, Qassim University, Buraydah 51452, Saudi Arabia; aladhadh@qu.edu.sa

**Keywords:** non-thermal processing, high-pressure processing, pulsed electric fields, cold plasma, process optimization, food safety, sustainability, Industry 4.0, ultrasound, digital twin

## Abstract

This is Part II of a two-part critical review of smart egg processing: the integration of sensing, artificial intelligence (AI), and processing technologies into one connected system. Building on Part I (non-destructive sensing and AI-based quality prediction), it reviews non-thermal egg processing technologies (high-pressure processing, pulsed electric fields, ultrasound, cold plasma, and ultraviolet treatments), AI-based parameter optimization, and effects on nutritional and functional quality. It then examines requirements for an integrated architecture: closed-loop control, digital twins and traceability, food safety, sustainability, scale-up, and standardization. Each technology is a mechanistically distinct but individually limited route to microbial inactivation. In the laboratory-scale egg studies reviewed, hurdle combinations at moderate intensity generally outperformed single technologies, although differences in matrices, organisms, and conditions preclude direct comparison. One laboratory study applied AI-driven multi-objective optimization to ultrasound–mild-heat co-sterilization of liquid whole egg, improving microbial safety and foaming retention together. In the literature searched, no study linked real-time sensing to automated non-thermal process control for eggs beyond the conceptual stage, and measured egg-specific energy, life cycle, and economic data were largely absent. A knowledge gap analysis, research agenda, and evidence-tiered conceptual framework (Smart Egg Processing 4.0) are presented.

## 1. Introduction

This paper is Part II of a two-part critical review examining **smart egg processing**: the integrated use of advanced sensing, artificial intelligence and machine learning, and non-thermal or minimally invasive processing technologies, coordinated through real-time data flows, to achieve quality grading, safety verification, and process optimization simultaneously rather than sequentially. Part I [1] of this review established the sensing and prediction half of that definition in detail: it evaluated nine non-destructive sensing technologies for eggs (computer vision, hyperspectral, and multispectral imaging; near-infrared, Raman, and fluorescence spectroscopy; electronic nose sensing and biosensors), the classical machine learning and deep learning methods used to interpret their output, and how those methods have been applied to specific quality and safety prediction tasks (freshness, defect detection, yolk characteristics, spoilage, and authentication).

Three findings from Part I [1] carry forward directly into this paper and are restated briefly because the arguments in this paper depend on them. First, non-destructive sensing for eggs is a mechanistically diverse and reasonably mature field, but the sensing technologies reviewed cluster unevenly across a distinction that matters throughout this paper as well: some technologies (notably computer vision and biosensors) measure a quality or safety attribute directly, while most others (hyperspectral and multispectral imaging, spectroscopy, electronic nose sensing) infer it from a correlated physical or chemical proxy. Second, machine learning and deep learning models applied to egg quality and safety prediction report frequently high accuracy, but that accuracy is overwhelmingly derived from internal cross-validation rather than testing against genuinely independent facilities, flocks, or seasons; the single study in the egg literature that tested this directly found a model’s internal accuracy ranking inverted entirely against real-world validation samples. Third, no study identified in either part of this review connects real-time sensing directly to an automated processing decision, a gap this paper examines from the processing side just as Part I [1] examined it from the sensing side.

This paper takes up the second half of the smart egg processing pathway. It critically reviews non-thermal processing technologies for eggs, high-pressure processing, pulsed electric fields, ultrasound, cold plasma, and ultraviolet and pulsed light treatments (Section 2), and the emerging, largely egg-specific-evidence-limited application of artificial intelligence to optimize their operating parameters (Section 3). It examines the effects of these technologies, individually and in combination, on egg protein structure, digestibility, bioactive compounds, lipids, and the applied functional properties (foaming, gelling, and emulsifying capacity) that give eggs much of their commercial value (Section 4). It then moves to the systems-level requirements a genuinely integrated smart egg processing architecture would need: real-time process monitoring and closed-loop control (Section 5), digital twins and traceability (Section 6), food safety and risk management (Section 7), sustainability and resource efficiency (Section 8), industrial scale-up and economic feasibility (Section 9), and standardization, validation, and regulatory challenges (Section 10). The paper closes by bringing these threads together with the findings of Part I [1]. It sets out a structured account of the field’s knowledge gaps (Section 11) and a timetabled future research agenda (Section 12). It then presents a conceptual framework that synthesizes the evidence from both papers into a single architecture, with each component explicitly classified by evidence tier (Section 13), and ends with overall conclusions for the two-part review (Section 14). The central thesis of Part II is that individual non-thermal and AI technologies are reasonably mature in isolation, whereas the field’s defining bottleneck is the lack of an empirically validated connective architecture linking them: sensing, prediction, process selection, and verification have each been demonstrated separately, but not as an integrated, closed-loop system for eggs.

Section numbering in this paper is independent of Part I: Part I [1] comprises Sections 1–5, and this paper is numbered from Section 1. Cross-references to Part I [1] are always marked “(Part I)” (for example, “Section 4.4 (Part I) [1]”), whereas unmarked references refer to sections of this paper. Wherever an argument depends on a specific finding from Part I [1], the dependency is cited explicitly and not assumed. The same evidentiary standard applies across both papers. Findings are characterized as laboratory proof-of-concept, pilot-scale, or industrially validated wherever the underlying literature allows that distinction, and evidence from non-egg food systems is flagged as such rather than presented as if it applied directly to eggs. Direct egg-specific evidence is distinguished from conceptual extrapolation throughout: statements supported by egg-specific studies are cited to those studies, and extrapolations from other food systems are labeled as such.

Literature search and evidence classification. This review is narrative and critical rather than systematic. The literature base was supplemented and verified in October 2026 through targeted searches of PubMed and a general web search engine, supplemented by targeted searches in Google Scholar and regulatory portals (FDA, USDA-FSIS, EFSA) for standards and guidelines (last search: 19 October 2026). Search terms were drawn from the following clusters and applied to each topic in combination with egg-related terms (egg, liquid whole egg, egg white, egg yolk, eggshell): non-thermal processing (high-pressure processing, pulsed electric field, ultrasound, cold plasma, ultraviolet, pasteurization, *Salmonella*, functional properties), AI and optimization (artificial intelligence, machine learning, genetic algorithm, response surface methodology, process optimization), systems integration (closed-loop, real-time control, digital twin, traceability, blockchain), and sustainability (life cycle assessment, energy consumption, carbon footprint). A 2022–2026 date filter was applied to the PubMed searches for recent studies, and earlier primary studies were retrieved by title or author. Official statistics, regulations, and standards were taken from the issuing bodies. Studies were included when they reported egg-specific experimental or modeling data; evidence from other food systems was used only as context and is labeled as such.

Statements of absence in this paper (for example, “no study was identified” or “not demonstrated”) refer only to the literature retrieved by this search. They indicate that egg-specific evidence was not found, not that it cannot exist, and they may change as new studies appear. Most egg-specific evidence reviewed here comes from laboratory-scale, single-facility experiments; no egg-specific industrial validation of AI-optimized integrated non-thermal egg processing, real-time closed-loop sensing–processing systems, or long-term production-line operation of the integrated configurations examined here was identified; where individual non-thermal technologies such as HPP have reached commercial deployment in food processing generally, this does not extend to the AI-optimized or fully integrated sensing–processing systems this review examines, and direct comparison among studies is limited by differences in microorganisms, matrices, processing conditions, and evaluation criteria.

## 2. Non-Thermal Technologies for Egg Processing and Preservation

Conventional thermal pasteurization of shell and liquid egg products kills microorganisms by denaturing their proteins. The same heat that inactivates *Salmonella* also denatures the egg’s own functional proteins, which is why pasteurization schedules for egg products are tightly bounded: low temperature and extended holding time, chosen to stay just below the coagulation threshold [2]. Non-thermal technologies are attractive in this context because they act through mechanisms that do not depend on bulk heating, namely mechanical disruption, electroporation, reactive-species oxidation, and photochemical damage. In theory, this gives them the potential to decouple microbial safety from thermal protein damage. Whether that potential is realized, and to what degree, differs substantially across the five technology families reviewed in this section. We therefore deliberately avoid treating them as a single undifferentiated category of “non-thermal alternatives,” because their mechanisms, and with them their egg-specific strengths and weaknesses, are quite distinct. The term “non-thermal” is also used with caution: several of the ultrasound and PEF studies below operate at 50–55 °C, so those treatments are better described as mild-heat assisted (for example, thermosonication) than as purely athermal.

### 2.1. High Hydrostatic Pressure Processing

High hydrostatic pressure processing (HPP) inactivates microorganisms mainly by disrupting cell membranes and denaturing microbial proteins, at pressures typically between 100 and 600 MPa and without the bulk temperature rise of thermal pasteurization. In liquid whole egg, the central practical difficulty is that egg proteins are themselves pressure sensitive. Purified ovotransferrin begins to aggregate partially at pressures above about 200 MPa (pH 8), with loss of sulfhydryl groups and increased surface hydrophobicity [3], and albumen proteins in general unfold and aggregate at pressures below those needed for reliable *Salmonella* inactivation. The treatment intensity that widens the safety margin therefore also risks the coagulation and viscosity changes that pasteurization is meant to avoid in the first place. A comprehensive review of HPP in liquid whole egg addresses this tension directly. Treatment at 300 to 450 MPa and 0 to 50 °C for up to 30 min reliably inactivates a range of bacteria in liquid whole egg. To avoid protein coagulation, however, the review recommends pressures of 300 MPa or less, temperatures of 20 °C or less, and holding times of five minutes or less, and it states that these conditions are not sufficient for full pasteurization on their own [2]. This is a mechanistically grounded trade-off, not a simple question of dose: antimicrobial efficacy and the risk to functional properties both scale with the same pressure and temperature parameters. That is why the reviewed literature has moved toward combination approaches, such as pairing HPP with antimicrobial compounds like nisin or with a second non-thermal or mild thermal step, instead of relying on HPP alone at higher intensities.

High-pressure homogenization (HPH) is related to HPP but mechanistically distinct, since it combines elevated pressure with intense shear as the product passes through a narrow valve. It has yielded some of the more detailed egg-specific functional-property data available. Multi-pass HPH of liquid whole egg at 100 MPa produced first-order inactivation kinetics against *Salmonella enterica* serovar *Senftenberg*, whereas equivalent thermal treatment at 65 °C gave non-linear survival curves with tailing. The same body of work found that HPH at 150 MPa modified egg white protein structure, inducing unfolding and aggregation through hydrophobic interactions. This reduced immunoreactivity, plausibly through epitope masking during aggregation, without a corresponding loss of foaming capacity [4]. The combination is instructive: a genuine structural change in the protein coexisted with a preserved functional property that is directly relevant to the commercial value of egg white. It shows why functional property assessment needs to be attribute-specific and not treated as a single “quality” outcome. A processing condition can measurably alter protein structure while leaving intact the functional property that a bakery or confectionery customer actually cares about.

### 2.2. Pulsed Electric Fields

Pulsed electric field (PEF) processing applies short, high-voltage pulses to a liquid product and inactivates microorganisms mainly through electroporation, the formation of pores in the microbial cell membrane under the applied field. Among the technologies reviewed in this section, PEF has the most directly documented egg-specific limitation. The comparatively high viscosity of liquid whole egg has been reported to blunt PEF’s antimicrobial effectiveness substantially in at least one controlled comparison. Huang et al. [5] tested PEF, high hydrostatic pressure, and ultrasound side by side against *Salmonella enteritidis* in liquid whole egg. Even at their optimized PEF condition of 30 pulses at 5.67 kV/mm and 55 °C, PEF was not effective. The authors attributed this explicitly to the high viscosity of the egg matrix, which limits how the electric field can be distributed through the product. High hydrostatic pressure and ultrasound, particularly in combination, achieved a meaningfully greater reduction under the same experimental framework, with 3.2 log cycles for the best combined treatment [5].

This finding should not be read as a blanket dismissal of PEF for egg applications. A dedicated review of PEF processing for egg products documents a more nuanced picture. Other studies, working with lower-viscosity egg fractions or different pulse configurations, have reported meaningfully better outcomes. These include a 3.5-log reduction in viable *S. enteritidis* in egg white using nine exponential-decay pulses at 35 kV/cm, and synergistic inactivation of *Escherichia coli* O157:H7 and *S. enteritidis* in liquid egg yolk when PEF was combined with elevated temperature [6]. The pattern across this literature is instructive because it resolves an apparent contradiction instead of leaving it unexamined. PEF’s effectiveness against egg-associated pathogens depends heavily on three things: the egg fraction treated (the viscosity of whole egg is a genuine obstacle that egg white or yolk alone may not present to the same degree), the electric field strength and pulse configuration, and whether PEF is used alone or as one step in a hurdle approach combined with mild heat or another non-thermal method. The same PEF-specific review also documents a distinct mechanistic effect that bears on the discussion of functional preservation in Section 4. PEF exposure has been reported to induce partial unfolding of ovalbumin and altered sulfhydryl-group reactivity. In at least one line of evidence, this structural change was associated with enhanced antioxidant activity in the resulting egg-derived peptides. PEF-induced protein modification is therefore not uniformly a liability. In specific cases it can generate a functionally desirable outcome, not merely a quality defect to be minimized [6]. The conditions of these studies are compared side by side in Section 2.6. Differences in matrix (whole egg, albumen, yolk), pulse waveform and treatment time, and temperature, rather than field strength alone, most plausibly explain the divergent outcomes: the whole egg study that found PEF ineffective used a higher nominal field (5.67 kV/mm, equal to 56.7 kV/cm) than the egg white study that reported a 3.5-log reduction (35 kV/cm), and the whole egg studies that reported larger reductions combined PEF with mild heat [6,7]. In whole egg, field strengths of 19 and 32 kV/cm preserved foaming capacity relative to untreated egg, whereas 37 kV/cm reduced it [8].

### 2.3. Ultrasound

Power ultrasound inactivates microorganisms mainly through acoustic cavitation, the formation and violent collapse of microscopic bubbles, which generates localized shear forces, heat, and free radicals capable of damaging microbial cell walls and membranes. In the same three-way comparison discussed in Section 2.2, ultrasound at 40 W and 55 °C for five minutes was one of the two effective single technologies against *S. enteritidis* in liquid whole egg, alongside high hydrostatic pressure. Longer exposure and higher temperature both improved its effectiveness. Combining ultrasound with high hydrostatic pressure gave the strongest reduction in that study, 3.2 log cycles, but the authors explicitly describe the combination as additive and not synergistic [5]. This distinction should not be collapsed into a generic statement that “combination works better.” It indicates that the two technologies inactivate largely independent sub-populations, or act through mechanisms that do not interact, and do not potentiate one another. Ultrasound’s dependence on temperature also places it, in practice, closer to a mild thermal-assisted technology than to a purely athermal one. Most reported egg applications pair ultrasound with a moderate temperature increase, typically in the 50 to 55 °C range, and do not use it at ambient temperature alone. This is a meaningful qualification when comparing ultrasound’s reported efficacy with that of more genuinely athermal technologies, such as high hydrostatic pressure at low temperature. Accordingly, “ultrasound–mild heat” or thermosonication is a more accurate label than “non-thermal” for these treatments, and ultrasound is grouped with the non-thermal family here only in the conventional sense.

### 2.4. Cold Plasma

Cold (non-thermal) atmospheric plasma is a partially ionized gas generated at or near ambient temperature. It contains reactive oxygen and nitrogen species, ozone, and, depending on the configuration, UV photons. It inactivates surface microorganisms mainly through the oxidative damage that these reactive species cause to cell membranes and macromolecules. Because it is inherently a surface treatment, cold plasma has been studied for eggs almost exclusively as a shell decontamination technology rather than an internal pasteurization one, which distinguishes it functionally from the liquid egg-focused HPP, PEF, and ultrasound literature discussed above.

Reported microbial log reductions for cold plasma applied to eggshells vary considerably across studies, from roughly 1 log to more than 7 log CFU per egg, and that spread is itself the more informative finding than any single figure, because the variation tracks specific, identifiable process, and biological variables rather than appearing as unexplained noise. Wan et al., using high-voltage atmospheric cold plasma (85 kV) in a modified atmospheric gas environment, achieved a 5.53 log CFU/egg reduction in *S. enteritidis* after 15 min of direct treatment, with no significant difference in egg quality parameters between direct and indirect exposure modes [9]. A separately reported cold atmospheric pressure air plasma jet achieved a substantially larger reduction, greater than 7 log_10_ CFU/egg after 120 s, while leaving eggshell cuticle integrity intact by both electron microscopy and infrared spectroscopy assessment, a mechanistically important finding since cuticle preservation is directly relevant to the shell’s own antimicrobial barrier function discussed in Section 2.1 (Part I) [1]. Dasan et al., working with non-thermal atmospheric plasma for eggshell surface decontamination more broadly, add a further, biologically grounded source of variability to this picture: bacterial physiological state measurably affects plasma susceptibility, with long-term survival phase *Salmonella* cells exhibiting substantially greater tolerance to cold plasma treatment than stationary phase cells [10], which means a reported log reduction obtained with a freshly grown laboratory inoculum may not directly predict performance against the more physiologically heterogeneous bacterial populations present on eggs under real storage and handling conditions. Treatment time, direct versus indirect exposure geometry, feed gas composition and humidity, applied voltage or power, and bacterial physiological state together account for most of the spread across these studies, which argues against treating cold plasma’s reported efficacy as a single number and in favor of the process parameter-dependent framing we apply throughout Section 2.

### 2.5. Ultraviolet and Pulsed Light Technologies

Ultraviolet-C (UV-C) and pulsed-light technologies inactivate microorganisms primarily through direct photochemical damage to microbial DNA, and, like cold plasma, have been applied to eggs in two functionally distinct modes: continuous or pulsed UV treatment of the intact eggshell surface, and UV-C treatment of liquid egg products passed through a thin-film or coiled-tube reactor designed to maximize UV penetration through an otherwise strongly UV-absorbing liquid. On the shell side, pulsed UV light treatment of eggshells inoculated with *S. enteritidis* has been shown to achieve meaningful pathogen reduction while the same body of work has evaluated effects on shell and internal quality parameters in parallel [11,12], and a more recent study using continuous UV-C treatment reported concurrent *Salmonella typhimurium* reduction alongside measurable changes in yolk carotenoid content and fatty acid composition, a finding directly relevant to Section 4’s nutritional preservation question because it demonstrates that even a nominally surface-limited UV treatment can be associated with detectable compositional change in the yolk, whether through limited UV penetration, secondary oxidative effects, or storage condition interactions the original study was designed to disentangle [13]. A related advanced oxidation approach, a gas phase hydroxyl-radical process combining UV-C, hydrogen peroxide mist, and ozone, was reported to achieve more than a 5 log CFU/egg reduction of *S. enteritidis* with a 10 s treatment, compared with 2.76 log CFU/egg for a conventional sodium hydroxide–chlorine wash, without adverse effects on egg quality metrics or cuticle integrity over 35 days [14].

On the liquid egg side, UV-C treatment has been shown to reduce *S. enteritidis* in liquid egg products when delivered through engineered reactors that maximize light penetration through a thin, turbulent product layer [15]. A companion study used a coiled-tube UV-C reactor and specifically evaluated the functional and nutritional composition of the treated product. It reported that the technology has potential as a pre-treatment step that could lower the intensity of a subsequent mild heat pasteurization step [16]. This is the hurdle approach logic that recurs across nearly every non-thermal technology discussed in this section. The fundamental physical limitation of UV-C in liquid egg is optical, not microbiological. Egg proteins, lipids, and pigments absorb UV-C strongly, which limits penetration depth. Thin-film and coiled-tube reactor designs exist to solve exactly this engineering problem. Simply raising the UV dose does not overcome it, because a large fraction of the extra dose would still be absorbed within the same shallow surface layer and would not reach deeper into the product.

### 2.6. Cross-Technology Synthesis

Two conclusions emerge from reading Section 2.1, Section 2.2, Section 2.3, Section 2.4 and Section 2.5 together rather than in isolation. The first is that no single non-thermal technology reviewed here is uniformly superior to thermal pasteurization or to the other non-thermal technologies. Each carries a specific, mechanistically grounded limitation. For HPP and HPH, the functional property trade-off scales with the same parameters that drive microbial inactivation. For PEF, the documented problem is reduced effectiveness in whole liquid egg, linked to its viscosity. Ultrasound depends in practice on moderate heating to work fully, cold plasma is restricted to surface treatment and cannot reach the interior, and UV-C is limited by how far light penetrates a liquid product. Treating any one of them as a categorical replacement for thermal pasteurization would overstate the evidence reviewed here. Second, for most of the technologies discussed in this section, the combination or hurdle approach literature (non-thermal treatment paired with mild heat, an antimicrobial compound, or a second non-thermal technology) generally reports better outcomes than a single technology at moderate, quality preserving intensity, a pattern that recurs across independently conducted but heterogeneous studies, HPP with nisin, ultrasound with high pressure, PEF with heat, UV-C as a pasteurization pre-treatment, that it constitutes one of the more defensible general conclusions we can draw about non-thermal egg processing as a whole. That conclusion sets up a natural question we have not yet addressed: if the answer to preserving both safety and functional quality lies in combining and tuning multiple process parameters simultaneously rather than maximizing any single one, that is precisely the kind of multi-parameter optimization problem the AI and machine learning methods surveyed in Sections 4 and 5 (Part I) [1] are, in principle, well suited to address, a connection Section 3 takes up directly. The distinction drawn in Section 2.3 between additive and synergistic combinations carries forward: ultrasound combined with HPP was additive, indicating largely independent inactivation of sub-populations, whereas PEF combined with heat or nisin has been described as synergistic [6]. Multi-technology optimization (Section 3) must therefore establish whether combined hurdles act additively or synergistically, because additive pairs can be optimized with independent factor terms, whereas synergistic pairs require interaction terms in the response surface or surrogate model. With few exceptions, the egg-specific studies summarized in this section are laboratory or bench scale experiments, and direct comparison among them is limited by differences in microorganisms, matrices, processing conditions, and evaluation criteria. Table 1 summarizes the mechanism, demonstrated microbial and quality effects, and principal advantages and limitations of each non-thermal technology reviewed in this section. Table 2 compares the conditions of the PEF studies discussed in Section 2.2.

## 3. AI-Optimized Non-Thermal Processing

Section 2.6 closed on a specific question: if preserving both microbial safety and functional quality in egg processing depends on tuning several interacting process parameters simultaneously rather than maximizing any single one, is that a problem AI and machine learning methods can meaningfully help solve? This section answers that question as carefully as the evidence allows. The answer has two parts, which must be kept clearly separate. A small amount of genuinely egg-specific evidence exists, and it shows that the underlying concept works. A considerably larger body of evidence exists for AI-driven optimization of non-thermal processing in other food systems. That evidence is informative about what is conceptually possible, but it is not evidence about eggs, and we do not treat it as such.

### 3.1. The Optimization Pipeline, in Principle

**Figure 1:**
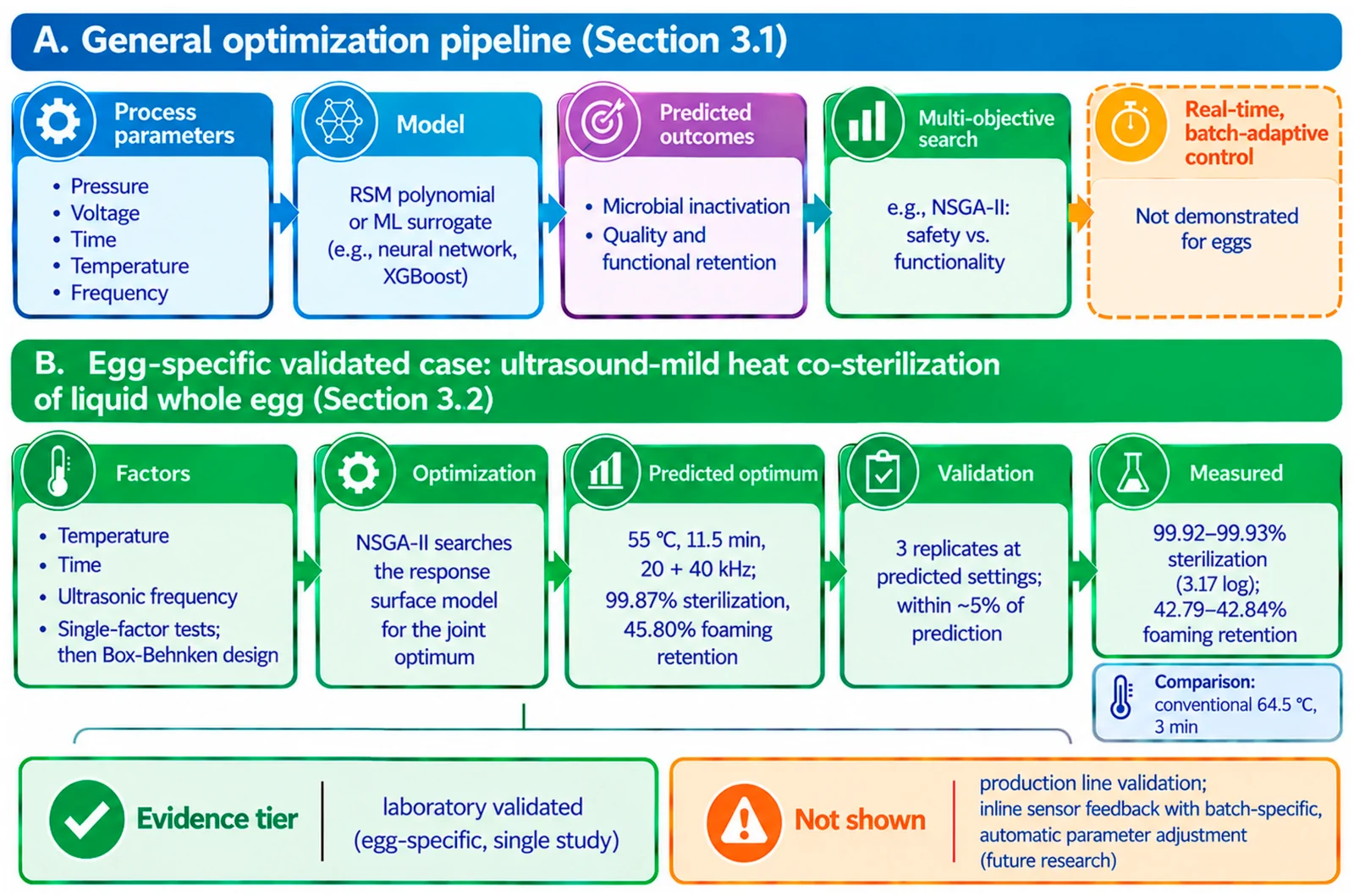
AI-assisted optimization of non-thermal processing. **Upper row**: general pipeline from process parameters through an RSM or machine learning model to predicted microbial and functional outcomes and multi-objective optimization; batch-adaptive real-time control is not demonstrated for eggs (Section 3.1). **Lower row**: the ultrasound–mild heat pipeline of Fan et al. [17] for liquid whole egg, validated at laboratory scale (Section 3.2). Original schematic prepared with Python 3.12.3 (Matplotlib 3.10.8); no generative image tools were used.

The general logic that links AI to non-thermal process optimization follows a consistent structure across the literature, egg-specific or otherwise. First, process parameters such as pressure, voltage, frequency, treatment time, and temperature are varied according to an experimental design. Second, a model is fitted to the relationship between those parameters and the outcomes that matter: microbial inactivation, a functional property retention metric, and energy consumption. The model may be a classical statistical response surface, a machine-learned regression, or a more complex algorithm. Third, an optimization algorithm searches the parameter space defined by that model and identifies the combination of settings that best satisfies the objectives, which often compete with one another. In the most advanced implementations, there is a fourth step: the optimized setting is applied and verified in real time, not fixed once and used indefinitely. Section 5 develops the real-time, closed-loop version of this pipeline in more depth; this section focuses on the optimization step itself; specifically, on what selects the parameter combination in the first place. Figure 1 (upper row) summarizes this general pipeline.

Terminology needs care here, because “AI-optimized” covers a wider range of methods than the supervised machine learning models discussed in Section 4 (Part I) [1]. Response surface methodology (RSM), a classical statistical technique for fitting a polynomial model to experimental data and identifying an optimum, has been used for decades in food process optimization and is not, in the strict sense, an AI method at all. Genetic algorithms and other evolutionary or metaheuristic optimization techniques, which search a parameter space guided by principles borrowed from natural selection rather than by learning from labeled training data, sit closer to the AI/ML domain in the broad sense adopted here, and are the method most directly relevant to the egg-specific evidence discussed next. True machine-learned predictive models of the kind discussed in Section 4 (Part I) [1], trained on process outcome data to predict microbial inactivation or quality retention as a function of process parameters, represent a further, more data intensive step beyond both RSM and evolutionary optimization. All three legitimately belong to the “AI-optimized processing” landscape that this section addresses, but they are not interchangeable. Evolutionary algorithms such as NSGA-II do not learn the relationship between parameters and outcomes; they need an objective (fitness) function to evaluate each candidate setting, which is supplied either by an RSM polynomial or by a trained machine learning surrogate (for example, a neural network or XGBoost) that predicts the outcomes for any parameter set. The surrogate predicts; the evolutionary algorithm searches. Conflating them would overstate how much of the egg-specific evidence below actually involves machine learning as Section 4 (Part I) [1] defined it.

### 3.2. Egg-Specific Evidence

One study identified in our literature search demonstrates the full pipeline described above applied directly to egg processing (Figure 1, lower row). It is described in some detail because it is, at present, close to the only example of its kind. Fan et al. [17] designed a synergistic ultrasound–mild heat sterilization system for liquid whole egg. They framed it explicitly around the tension we have emphasized throughout Section 2: the thermal intensity of conventional pasteurization threatens functional properties, while non-thermal treatment alone is often not effective enough [17]. The authors first established single-factor relationships between temperature, treatment time, and ultrasonic frequency on the one hand, and two outcome variables, sterilization rate and foaming property retention, on the other. They then fitted a Box–Behnken response surface design and applied a genetic algorithm, NSGA-II, to search the resulting parameter space for settings that would maximize sterilization rate and foaming property retention at the same time. NSGA-II is a non-dominated sorting genetic algorithm designed for problems with more than one, potentially competing, objective [17]. This is a materially different exercise from optimizing a single outcome. It treats the safety-versus-functionality trade-off that we have framed conceptually since Section 2.1 as a formal multi-objective optimization problem, instead of optimizing safety first and checking functional consequences afterward.

The result is instructive both for what it found and for how transparently it was validated. The genetic algorithm identified an optimum at 55 °C, 11 min 30 s, and combined 20 + 40 kHz multifrequency ultrasound, predicting a 99.87% sterilization rate and 45.80% foaming property retention [17]. The authors then ran physical validation experiments at exactly these predicted settings, in three replicate trials. The measured outcomes were a 99.92 to 99.93% sterilization rate, corresponding to a 3.17 log reduction, and 42.79 to 42.84% foaming retention. These landed within roughly 5% of the model’s predictions [17]. This 3.17-log reduction was the optimization study’s experimental target, benchmarked against China’s national pasteurization standard (64.5 °C for 3 min); it does not meet the more demanding regulatory thresholds applicable in other jurisdictions, such as the minimum 5-log *Salmonella* reduction required for liquid egg products under US USDA-FSIS regulations (9 C.F.R. Part 590) [18], and should be understood as a laboratory-validated optimization study outcome rather than as evidence of regulatory pasteurization compliance. Agreement of this kind between predicted and experimentally verified outcomes is a meaningful, if narrow, piece of evidence. It suggests that the optimization pipeline described in Section 3.1 can work as intended for at least one egg-processing application, and is not merely a theoretical construct. The authors also compared the optimized treatment directly with China’s national pasteurization standard for liquid egg (64.5 °C for 3 min). The optimized ultrasound thermal treatment achieved a comparable or superior foaming property outcome at a meaningfully lower peak temperature [17]. This result is consistent with the broader logic of hurdle-based non-thermal processing developed in Section 2.6.

This result should not be overstated. The authors’ own stated limitation is direct: the system was validated at laboratory scale and has not been applied to an actual liquid egg production line [17]. In the evidence hierarchy terms we apply throughout, this is laboratory scale proof-of-concept evidence. It is a genuine demonstration that the concept works under controlled conditions, but it is not evidence of pilot scale or industrial robustness. It is also, to reiterate the point made in Section 3.1, an application of a genetic algorithm to a response surface model rather than of the supervised machine learning methods discussed in Section 4 (Part I) [1], a distinction that matters because the AI-relatedness of a given technique should not be overstated. Figure 1 summarizes this pipeline, from the general optimization framework through the one validated egg-specific demonstration and its unresolved real-time connection.

### 3.3. Evidence from Other Food Systems

A substantially larger body of evidence addresses machine learning-driven optimization of non-thermal processing across food systems generally, and a comprehensive 2025 review synthesizes this landscape directly, covering high-pressure processing, pulsed light, ultrasound, pulsed electric fields, cold plasma, and irradiation [19]. None of the evidence synthesized in that review is egg-specific; it spans a wide range of other food matrices, and we cite it strictly as evidence of what has been demonstrated in food processing generally, not as evidence about eggs. Within that broader synthesis, several points are directly relevant to the egg-specific discussion above because they identify what a more data driven (as opposed to response surface- or genetic algorithm-based) approach could, in principle, add. The review reports that machine learning models have been developed specifically to predict microbial inactivation kinetics during high-pressure processing. This work is explicitly framed as an advance on earlier deterministic kinetic models, because it captures non-linear relationships between process parameters, food matrix properties, and inactivation outcomes that classical models struggle to represent [19]. The review’s own explanation of why this matters applies directly to the egg case, even though the underlying evidence does not come from eggs. Numerical, physics-based models offer deterministic insight into known mechanisms, but food matrices are complex and variable enough (as Section 2 has repeatedly emphasized for eggs specifically, in the context of whole egg viscosity’s effect on PEF performance) that a data driven model can reveal parameter interactions a fixed mechanistic equation would not capture [19].

### 3.4. Distinguishing What Is Known from What Is Plausible

Judged against the evidentiary standard applied throughout, the evidence on AI-optimized non-thermal processing of eggs can be summarized as follows. Egg-specific evidence exists for exactly one processing technology combination (ultrasound–mild heat co-sterilization of liquid whole egg) and one optimization method (a genetic algorithm applied to a response surface model), validated at laboratory scale with a single, encouraging round of experimental confirmation [17]. Evidence from other food systems demonstrates that more data-intensive machine learning approaches, applied to HPP, PEF, cold plasma, ultrasound, pulsed light, and irradiation, are an active and expanding area of general food process engineering research, with specific reported applications to microbial inactivation kinetics prediction [19]. Transferable concepts, meaning methodological approaches with a plausible mechanistic basis for working on eggs even though they have not yet been tested on eggs, include: multi-objective optimization treating safety and functional property retention as simultaneous rather than sequential goals, exactly as Fan et al. demonstrated for one technology pairing [17]; machine-learned (rather than purely response surface-based) predictive models of the kind reviewed by Rashvand et al., which could in principle be applied to the other non-thermal technologies discussed in Section 2, HPP, PEF, cold plasma, and UV, none of which currently have an egg-specific optimization study analogous to Fan et al.’s ultrasound work; and integration of the sensing and prediction methods surveyed in Sections 3–5 (Part I) [1] as inputs to such an optimization loop, so that the process parameters selected could, in principle, be tailored to a specific incoming batch’s measured or predicted freshness and composition rather than fixed for all batches alike, the connective step the Introduction identified as its central contribution.

Future research opportunities follow directly from that gap. The most immediate is straightforward extension: applying the multi-objective optimization approach Fan et al. [17] demonstrated for ultrasound–thermal co-sterilization to the other non-thermal technologies reviewed in Section 2, HPP, PEF, and cold plasma each have at least the beginnings of a parameter response literature (Section 2.1, Section 2.2, Section 2.3 and Section 2.4) that a genetic algorithm or machine learning optimization layer could, in principle, be built on top of, but has not yet been. A second, more demanding opportunity is pilot scale validation of the one egg-specific result that does exist, moving Fan et al.’s [17] laboratory demonstration toward the production line testing the authors themselves identify as the necessary next step. A third is closing the loop between sensing and optimization directly: none of the AI-optimization evidence reviewed in this section, egg-specific or otherwise, incorporates real-time sensing input of the kind reviewed in Section 3 (Part I) [1] to adjust process parameters batch-by-batch rather than applying one fixed optimum to all batches, which is precisely the architecture Section 5 discusses next.

## 4. Preserving Egg Nutrition and Functionality

This section addresses a single question: can smart processing improve microbial safety while minimizing undesirable changes in nutritional and functional quality? Section 2 and Section 3 already supplied a substantial part of the answer, since functional property retention was the second objective in nearly every processing study discussed there, not an afterthought measured separately. This section draws that evidence together explicitly, organized by the biochemical target (protein structure and digestibility, bioactive peptides, lipids and antioxidant status, and the applied functional properties of foaming, gelling, and emulsifying) rather than by processing technology, and closes with a direct comparison of thermal, non-thermal, and hybrid processing.

### 4.1. Protein Structure and Digestibility

Every non-thermal technology reviewed in Section 2 acts, at the molecular level, on the same target: the folded, native structure of egg proteins. In albumen, these are principally ovalbumin, ovotransferrin, and lysozyme, and in yolk, the lipoprotein and phosvitin fractions. What differs across technologies is the mechanism of the structural perturbation. HPP and HPH act through mechanical shear and pressure, PEF through electroporation-associated field effects, and ultrasound through cavitation-associated shear and localized heating. Each mechanism produces a structurally distinct kind of unfolding or aggregation, not a generic, interchangeable “denaturation.” The distinction matters directly for digestibility. Protein digestibility depends on how accessible the peptide backbone is to gastrointestinal proteases, and partial unfolding can either help or hinder that access. It helps when it exposes new cleavage sites and hinders when it drives aggregation that buries them. Controlled thermal treatment studies of egg white protein illustrate this non-monotonic relationship clearly: egg white proteins treated at 65 °C showed the highest in vitro pepsin digestibility among the temperatures tested, while combined pepsin-plus-pancreatin digestibility increased significantly as cooking temperature rose, and a separate study working across a temperature range from 20 to 90 °C found that partial unfolding at an intermediate temperature (60 °C) produced measurably higher gastrointestinal digestibility and antioxidant activity than either the native or the more extensively heat-aggregated protein, depending on the pH condition under which the protein was held. These findings are drawn from thermal rather than non-thermal treatment specifically, but the underlying structural logic, that partial, controlled unfolding can improve protease accessibility while excessive aggregation can reduce it, is directly relevant to interpreting the HPH-induced immunoreactivity and foaming results discussed in Section 2.1, since immunoreactivity reduction via epitope masking during aggregation and preserved digestibility are not guaranteed to move together, and we are not aware of egg-specific non-thermal processing data that directly measures both endpoints in the same study.

### 4.2. Bioactive Peptides

Non-thermal processing’s relevance to bioactive peptide generation is primarily as a pre-treatment step ahead of enzymatic hydrolysis, rather than as a peptide-generating mechanism in its own right: ultrasound, HPP, and PEF are reported, across food proteins broadly, to improve the yield and bioactivity (antioxidant, angiotensin-converting enzyme-inhibitory, antimicrobial, and antidiabetic activity) of peptides subsequently released by enzymatic hydrolysis, through the same structural mechanism discussed in Section 4.1: conformational change that increases the protein’s susceptibility to protease cleavage [20]. This synthesis is drawn from a review that spans animal and plant protein sources in general, not from egg-specific evidence. It is cited here accordingly, as a mechanistic and conceptual frame and not as a direct egg finding. The egg-specific bioactive-peptide literature is more established on the enzymatic-hydrolysis side than on the non-thermal-pretreatment side: egg proteins are a well-documented source of peptides with antihypertensive (ACE-inhibitory), antioxidant, antimicrobial, anti-inflammatory, antidiabetic, and mineral-binding activity, generated primarily through simulated or applied gastrointestinal digestion and enzymatic hydrolysis [21]. Egg-specific antioxidant activity has also been traced to intact proteins themselves, ovalbumin, ovotransferrin, and lysozyme in albumen, and phosvitin in yolk, in addition to their derived peptides, with derived peptides generally showing higher bioactive potency than the intact parent proteins. Where this leaves the specific question motivating Section 3, whether non-thermal processing can be used deliberately to enhance bioactive peptide yield from eggs specifically, the conclusion follows the same structure as Section 3.4’s conclusion: the mechanistic rationale is sound and supported by evidence from other protein sources [20], but a dedicated body of egg-specific evidence testing non-thermal pre-treatment’s effect on egg-derived bioactive peptide yield and potency, as opposed to conventional heat treatment’s effect, was not identified in comparable depth for this review, and stands as a specific, well-defined research opportunity rather than an established finding.

### 4.3. Lipids and Antioxidant Activity

Yolk lipids, together with the carotenoid pigments (lutein and zeaxanthin) and fat-soluble vitamins associated with them, are the compositional attributes most directly documented as processing sensitive among the non-thermal studies reviewed in Section 2. The clearest single data point comes from the discussion of continuous UV-C treatment in Section 2.5. That study reported a reduction in *S. typhimurium* together with a measurable change in yolk carotenoid content and fatty acid composition [13]. A safety-oriented non-thermal treatment is thus linked to a nutritionally relevant compositional shift within one study, with no need to infer across separate literatures. PEF exposure has similarly been associated with partial unfolding of ovalbumin and altered sulfhydryl-group reactivity, changes that in at least one line of evidence were linked to enhanced, rather than diminished, antioxidant activity in resulting egg-derived peptides [6], a reminder, consistent with the framing in Section 2.6, that processing induced structural change is not intrinsically a quality loss and can in specific, mechanistically explicable cases generate a functionally desirable outcome. A plausible mechanism, offered here as an interpretation and not as a finding of the cited review, is that field-induced conformational change exposes buried hydrophobic residues and sulfhydryl groups: free thiols can donate electrons or hydrogen atoms to scavenge radicals, and exposed hydrophobic residues can interact with lipid radicals, so the structural alteration increases radical scavenging capacity instead of representing a quality loss. Taken together, the lipid- and antioxidant-relevant evidence reviewed across this manuscript supports a specific, moderate conclusion: non-thermal processing measurably interacts with yolk lipid and pigment chemistry, in directions that are technology- and condition-dependent rather than uniformly protective or uniformly damaging, and that interaction is detectable within the same studies that establish microbial efficacy rather than requiring separate confirmation, which is a genuine methodological strength of the egg-specific non-thermal literature relative to some of the sensing and AI literature reviewed in Sections 3–5 (Part I) [1], where safety and compositional endpoints are more often measured in separate studies entirely.

### 4.4. Functional Properties: Foaming, Gelling, and Emulsifying

Foaming, gelling, and emulsifying capacity are the functional properties with the most direct commercial relevance to egg products, since they determine performance in bakery, confectionery, and processed food applications, and they are also the properties most consistently measured alongside microbial efficacy in the non-thermal literature reviewed in this manuscript, which allows a genuinely comparative synthesis rather than a technology-by-technology recitation. HPH at 150 MPa altered egg white protein structure through hydrophobic interaction-driven unfolding and aggregation, reduced immunoreactivity, and left foaming capacity statistically unaffected [4], demonstrating that a measurable structural change need not translate into a commercially relevant functional loss. Fan et al.’s ultrasound–thermal co-sterilization system, discussed in detail in Section 3.2, found that foaming property first improved and then declined as ultrasound treatment time and temperature increased, with an optimum window (around 55 to 60 °C, moderate treatment time) beyond which foaming stability declined sharply, and, critically, that the optimized non-thermal hybrid treatment achieved foaming performance superior to a comparison pasteurization treatment at equivalent or better microbial safety [17]. UV-C treatment of liquid egg via engineered thin-film and coiled-tube reactors has similarly been evaluated specifically for its effect on functional and nutritional composition, with the technology’s proposed value framed explicitly around its potential to reduce the intensity of a subsequent thermal step, and by extension the thermal damage that step would otherwise cause to functional properties [16]. Gelling and emulsifying capacity were not reported in most of the non-thermal studies summarized in Table 3; “not reported” entries therefore indicate an absence of data in the cited sources, not evidence that these properties were tested and unaffected. Gelling and emulsifying properties have been studied for high-pressure and ultrasound modified egg proteins in the protein modification literature [22], but that work lies outside the safety-oriented studies summarized here. In whole egg, PEF at 19 and 32 kV/cm preserved foaming capacity, whereas 37 kV/cm and heat pasteurization at 66 °C for 4.5 min reduced it [8].

### 4.5. Comparative Synthesis: Thermal, Non-Thermal, and Optimized Hybrid Processing

Assembling the evidence from Section 2, Section 3 and Section 4.1, Section 4.2, Section 4.3 and Section 4.4 into a direct three-way comparison, conventional thermal pasteurization, single non-thermal technologies, and optimized hybrid (non-thermal-plus-mild-thermal) processing, produces a reasonably consistent picture, although it must be interpreted with caution because much of the underlying evidence rests on a comparatively small number of egg-specific studies. Conventional thermal pasteurization is the most extensively validated approach for microbial safety, but the same studies that establish its safety efficacy consistently document measurable functional property cost, protein coagulation risk, foaming and viscosity changes, that scales with the same temperature and time parameters driving microbial inactivation [2]. Single non-thermal technologies applied alone show a genuinely mixed record: HPP and HPH can achieve substantial inactivation with real but attribute-specific functional trade-offs [2,4]; PEF’s effectiveness in whole liquid egg specifically is constrained by the matrix’s viscosity in at least one controlled comparison [5]; ultrasound alone is explicitly not recommended as a standalone sterilization method by regulatory guidance cited within the egg-specific ultrasound literature, which is precisely why every ultrasound application reviewed in this manuscript pairs it with mild heat rather than deploying it alone [17]; and UV-C and cold plasma are fundamentally surface or thin-film limited, which bounds their applicability to shell decontamination and specially engineered liquid egg reactors respectively rather than general purpose pasteurization. Optimized hybrid processing is where the synthesized evidence points most consistently toward genuinely superior outcomes. It combines a non-thermal treatment with carefully tuned mild heat, an antimicrobial compound, or a second non-thermal technology, and is further tuned through the AI-assisted optimization approaches discussed in Section 3. The ultrasound-thermal system of Fan et al. achieved both a laboratory validated optimization study sterilization outcome and superior foaming performance relative to the conventional pasteurization comparison used in that study [17]. The broader hurdle technology pattern identified in Section 2.6 reflects the same underlying principle applied across several technology pairings: HPP with nisin, ultrasound with pressure, PEF with heat, and UV-C as a pre-treatment before pasteurization.

The same caveat applies to the processing literature. The comparative picture is built almost entirely from laboratory scale, single study, single facility evidence. The same validation gap that Section 4.4 (Part I) and Table 4 (Part I) [1] document rigorously for AI-based quality prediction applies with equal force to the processing literature synthesized in this section. A hybrid processing condition that outperforms conventional pasteurization in one laboratory’s foaming assay has not, on that evidence alone, been shown to do so reliably across the flock variability, seasonal variation, and equipment differences that a genuine industrial deployment would encounter. Section 9 returns to this limitation directly when the review turns from what the evidence shows to what would actually be required to deploy it. Table 3 consolidates the specific nutritional and functional effects documented across Section 2, Section 3 and Section 4 for each technology, together with the evidence tier that supports each finding.

## 5. Real-Time Process Monitoring and Closed-Loop Control

The sensing, prediction, and processing technologies surveyed across this two-part review add up to a genuinely “smart” system only if they are connected into a functioning loop and not operated as isolated steps. These are the sensors and prediction models of Part I [1] and the processing methods of Section 2, Section 3 and Section 4 of this paper. The framework we adopt for that loop, consistent with how process control engineering generally describes automated systems, is: sense, analyze, predict, decide, act, verify. A sensor (Section 3 (Part I) [1]) acquires a signal. An algorithm (Section 4 (Part I) [1]) analyzes it, and a model (Section 5 (Part I) [1]) predicts a quality or safety attribute. A decision rule then selects a processing action (Section 2 or Section 3), the action is applied, and the outcome is verified, ideally feeding back into the next cycle’s decision. This section asks a direct question of the egg-specific literature: how much of that loop has actually been built, as opposed to proposed?

### 5.1. What Has Been Demonstrated: Real-Time Sensing and Sorting

The strongest, most industrially credible evidence for real-time operation in this manuscript concerns the sensing-to-sorting portion of the loop, sense, analyze, decide (reject or grade), act (physically divert the egg), rather than the full loop through to process-parameter adjustment, drawing on both the sensing technologies reviewed in Part I [1] and the real-time systems evidence developed later in this section. Akkoyun et al.’s industrial multi-flow grading system is a clear example: a color camera mounted above a motorized roller conveyor classified eggs by weight in real time on an actual industrial production line, achieving a correlation of R^2^ = 0.95 between image-derived and measured weight [23], and the system’s own framing, as a deployed, operating industrial line rather than a laboratory prototype, places it at the more mature end of the evidence hierarchy we apply throughout. Chen et al.’s factory-oriented hyperspectral system, discussed in Section 3.3 (Part I) [1], was similarly framed around production line and consumer-facing deployment, reporting freshness and defect detection accuracies of 91% and 93%, respectively, using a multifunctional camera and XGBoost pipeline [24]. Wu et al.’s machine vision monitoring system, though implemented at the egg collection (henhouse) stage rather than at a downstream processing facility, and therefore adjacent to rather than squarely within our processing-focused scope, is described here because it demonstrates, with real infrastructure-level numbers, what a functioning sense-to-decision pipeline looks like end to end: an enhanced YOLOv8s object detection model deployed on a Jetson AGX Orin edge computing device achieved a precision of 94.0%, recall of 92.8%, and F1 score of 93.4% at 91.7 frames per second, with the system transmitting data over a wireless network (measured latency around 35 milliseconds) to cloud infrastructure for a real-time management dashboard, and field validation across 60 commercial chicken cages achieved a 98.9% egg recognition rate [25]. Although this system sits at the farm rather than the processing plant stage of the supply chain, the architecture it demonstrates, edge inference for latency sensitive detection, cloud infrastructure for aggregation and dashboarding, and a measured, sub-100 millisecond network latency, is directly the kind of infrastructure a processing line implementation of the sensing technologies reviewed in Section 3 (Part I) [1] would need, and no processing stage study identified in our literature search reports comparably detailed infrastructure metrics.

### 5.2. What Has Not Been Demonstrated: Closing the Loop Back to Process Parameters

**Figure 2:**
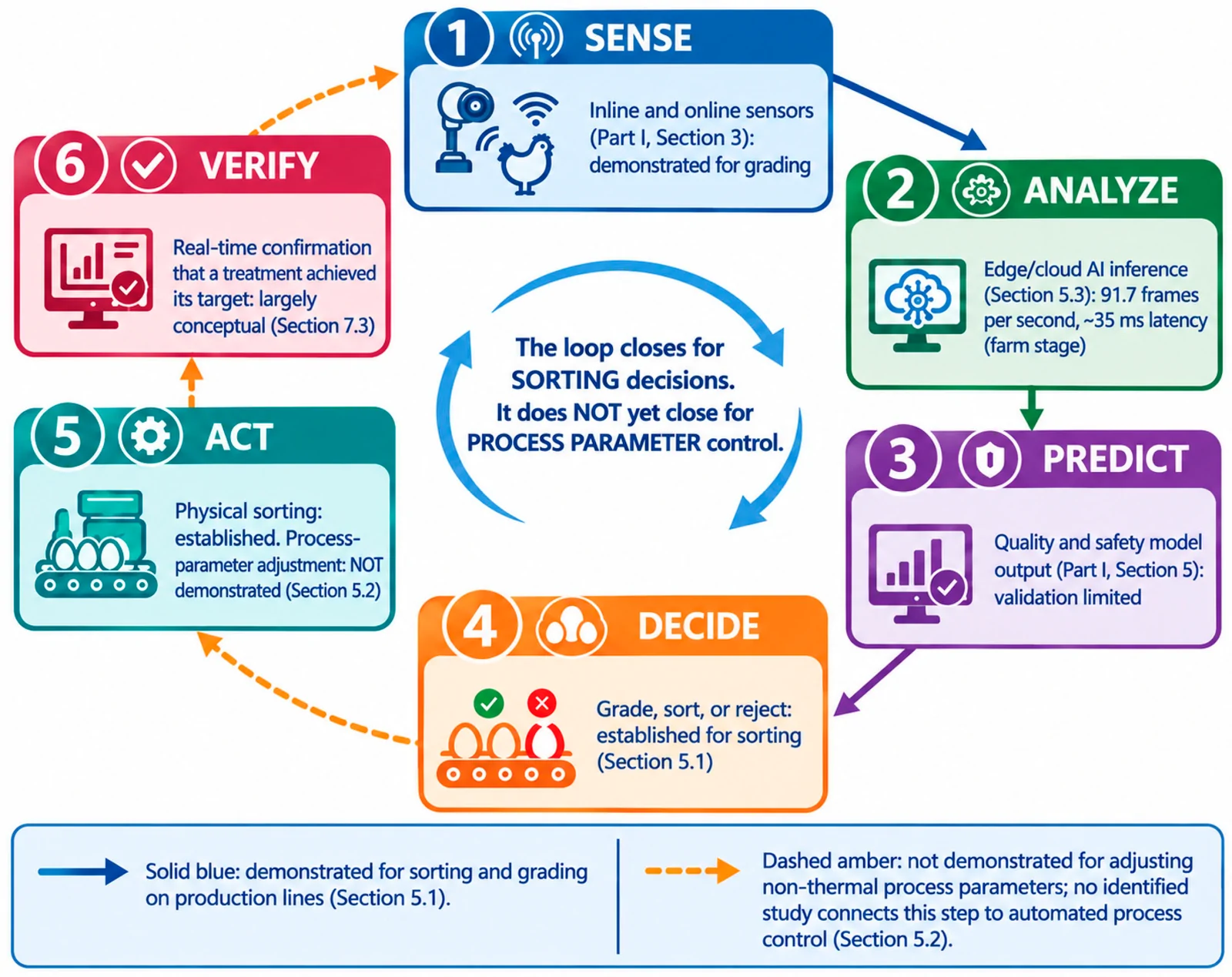
Closed-loop smart egg processing system: six-stage sense–analyze–predict–decide–act–verify loop. Solid arrows indicate segments demonstrated for sorting and grading on production lines (Section 5.1); dashed arrows indicate segments not demonstrated for adjusting non-thermal process parameters (Section 5.2). In the Act stage, physical sorting is established, whereas process-parameter adjustment has not been demonstrated; the two should not be conflated under the term “real-time processing”. The purple arrow indicates the transfer of prediction outputs to the decision stage. The shield represents quality and safety assessment, while the monitor and checkmark symbolize model-generated predictions, with validation remaining limited. ✓ Green checkmark “Accept”: Product meets the required quality criteria and is approved for grading/sorting. ✕ Red cros “Reject”: Product does not meet the required quality criteria and is rejected. They illustrate the classification outcomes of the sorting decision process. Original schematic prepared with Python 3.12.3 (Matplotlib 3.10.8).

Here the evidence becomes noticeably thinner, and capability that has not been shown is not inferred. Every real-time system described in Section 5.1 closes its loop at the decision-and-act stage in the narrow sense of sorting, grading, or flagging an individual egg, not in the broader sense of adjusting a non-thermal processing parameter (pressure, voltage, ultrasonic frequency, treatment time) in response to what the sensor detected. Section 3 already established that exactly one egg-specific study demonstrates AI-driven optimization of non-thermal processing parameters, Fan et al.’s genetic algorithm-optimized ultrasound–thermal system [17], and that study optimized a single, fixed set of process parameters offline, applied uniformly to a batch, rather than adjusting parameters in real time in response to an inline sensor reading of that specific batch’s condition. No study identified in our literature search couples a real-time sensor of the kind reviewed in Section 3 (Part I) [1] directly to a non-thermal process controller of the kind reviewed in Section 2, such that, for example, a batch of liquid egg measured as compositionally atypical by inline NIR spectroscopy would automatically trigger an adjusted ultrasonic–thermal treatment intensity rather than the same fixed protocol applied to every batch. This is the “predictive and preventive process control” that would move processing beyond end-product inspection. On the evidence surveyed here, it remains a conceptually well-motivated but empirically undemonstrated target for egg processing specifically, and not an established capability that we can report as already achieved. This asymmetry is shown schematically in Figure 2, which distinguishes the demonstrated sensing to sorting segment (solid) from the undemonstrated process control segment (dashed).

A closed loop of this kind also requires fail-safe default logic, a standard principle in control engineering under which a fault drives the system toward a safe state instead of an unsafe one [26]. If an inline sensor fails, encounters an uncalibrated matrix anomaly, or drifts, the controller should detect the condition (for example, through signal range checks, drift monitoring, and out-of-distribution detection on the input data), revert automatically to a verified, conservative baseline such as the validated conventional thermal pasteurization schedule, and flag the affected batch for confirmatory microbiological testing. Redundant or orthogonal sensors and a defined hold-and-divert rule for uncertain batches would be needed so that no automated parameter adjustment is accepted unless the sensing signal itself passes validity checks.

### 5.3. Edge Computing, Cloud Computing, and the Latency Trade-Off

The choice between edge and cloud computing, evident in the example of Wu et al. [25] above, reflects a genuine engineering trade-off and not a simple preference. Edge deployment runs inference locally, on hardware at or near the sensing point. It minimizes the latency between acquiring a signal and acting on it. This matters directly for any application, such as sorting or defect rejection, in which the physical egg passes the sensor at line speed. There the decision window is measured in milliseconds, not seconds, and it determines whether the action can still be applied to that specific egg. The detection rate of 91.7 frames per second and the network latency of roughly 35 milliseconds reported by Wu et al. illustrate the order of magnitude that such applications require [25]. Cloud computing, by contrast, offers computational capacity and storage that a local edge device cannot match. This is why the same system paired edge inference for the time-critical detection task with cloud infrastructure for the less time-critical functions of dashboards, data aggregation, and historical records [25]. This division of labor, edge for latency sensitive inference, cloud for aggregation, dashboarding, and longer-term analysis, is the pattern we expect a mature real-time egg processing architecture to follow, based on the one detailed example available, although this example is drawn from the farm rather than the processing plant stage of the supply chain, and a processing line-specific demonstration of the same division of labor was not identified in our literature search.

A further, more specifically egg-relevant obstacle to real-time deployment was already noted in the hyperspectral imaging-specific literature discussed in Section 3.2 (Part I) [1]: calibration transfer between instruments and facilities remains a documented, unresolved challenge, with biological variability in the eggs themselves (hen strain, diet, storage history) capable of degrading a model’s accuracy when applied outside the conditions it was calibrated under, and the transition from offline laboratory calibration to real-time online calibration is explicitly identified as encountering data processing and prediction-timing challenges beyond those present in an offline setting [27]. This is not a hypothetical concern. It is the direct, practical form that the model validation gap documented in Section 4.4 (Part I) and Table 4 (Part I) [1] takes once a system moves from a laboratory bench toward a production line. Even the sensing technologies with the strongest offline accuracy figures reviewed in Sections 3–5 (Part I) [1] would need dedicated real-time recalibration infrastructure before the loop described in the introduction to Section 5.1 could close reliably at industrial scale. Faster hardware alone would not be enough.

## 6. Digital Twins, Traceability, and Smart Egg Processing Systems

### 6.1. Digital Twins

A digital twin, in the sense the food processing literature generally uses the term, is a continuously updated virtual representation of a physical system: a piece of equipment, a production line, or an entire facility. It is built from real-time sensor data and can simulate how the system would respond to a proposed change before that change is applied physically. Applied to egg processing, the concept would extend directly from the sensing and AI infrastructure reviewed in Sections 3–5 (Part I) [1] and the real-time architecture reviewed in Section 5. A digital twin of a liquid egg pasteurization line, for instance, could in principle simulate the effect of an adjusted non-thermal processing parameter on both microbial safety and functional property retention before an actual physical batch is committed to that treatment. This would extend the optimization logic that Section 3 demonstrated offline into a continuously updated, real-time simulation.

The evidence indicates that this remains substantially more concept than demonstrated capability, in food processing generally and in egg processing specifically. A 2025 review of digital twin applications across the food industry states directly that, because the technology is relatively new, research on its application within food processing remains limited. The review reached this conclusion after surveying the available literature, not as a caveat added defensively [28]. Egg-specific evidence is thinner still: the most direct egg-relevant discussion of digital twins identified in our literature search appears not as an independent case study but as a forward-looking conceptual proposal within the broader Industry 4.0 sensing review discussed throughout this manuscript, which describes a digital twin as a cyber-physical interface capable of remotely controlling poultry farm and egg processing operations by integrating sensing, monitoring, analysis, planning, and control, and explicitly frames this as a “next phase” or “next generation” capability rather than a system that has been built and evaluated [29]. We treat that description accordingly: as a well-reasoned articulation of what a digital twin for egg processing could look like, drawing on genuine building blocks (the sensing, AI, and real-time infrastructure reviewed in Sections 3 and 4 (Part I) [1] and Section 5 all being necessary components), not as evidence that such a system has been implemented or validated for eggs at any evidence tier beyond the conceptual.

### 6.2. Digital Traceability and Blockchain

Digital traceability is the tracking of an egg or batch of egg product’s identity, origin, and processing history through the supply chain. It is a more mature need than the digital twin concept discussed above, and one driven more directly by regulation. It is also an area where blockchain should be adopted only if it offers a genuine advantage, not because the technology is fashionable. The most directly relevant egg-specific evidence is a theoretical design study. It proposes a permissioned blockchain architecture that combines a proof-of-authority consensus mechanism with soulbound (non-transferable) tokens issued by regulatory authorities, specifically to track compliance with the European Union’s organic egg production and labeling regulation (EU 2018/848) [30]. The proposal deserves engagement on its merits and not outright dismissal, because it identifies a specific, genuine limitation of conventional traceability approaches. Attributes such as production system (caged, barn, free-range, organic) cannot be verified analytically from the egg itself, which is exactly the authentication problem discussed at length in Section 5.6 (Part I) [1]. For such attributes, a certificate-based traceability chain is one of the only mechanisms available to regulators for verifying compliance. Blockchain’s technical properties, immutability and multi-party verifiability without requiring every participant to trust a single central database operator, are a plausible fit for a supply chain of farm, packer, distributor, retailer, and regulator in which no single actor is inherently trusted by all the others [30].

That said, our assessment is that the case for blockchain specifically, as opposed to traceability and digital record-keeping more generally, rests heavily on that multi-party, low-trust condition. It is considerably weaker where the condition does not hold. A permissioned blockchain restricted to a small set of known, vetted participants, which is what the proposed EU organic egg design itself specifies, functions in practice more like a shared, cryptographically verified database among trusted or semi-trusted parties than like the fully decentralized, trustless architecture that blockchain is often marketed around. In that configuration, a well-designed centralized or federated database with appropriate access controls and audit logging can achieve much of the same traceability and tamper evidence with substantially less computational overhead and infrastructure complexity. The proposal is explicitly framed as a theoretical design and not as an implemented or piloted system [30]. We are not aware of an operational, deployed blockchain traceability system evaluated specifically for egg processing at any evidence tier beyond the conceptual. The defensible conclusion is that such technologies should be adopted only where they provide a real advantage. Digital traceability is a well-established and clearly beneficial goal for smart egg processing. Blockchain is one credible architectural option for achieving it in specific multi-stakeholder, low-mutual-trust configurations, such as cross-border organic certification. Its advantage over simpler centralized alternatives narrows considerably outside that condition. The egg-specific literature surveyed here draws this distinction more carefully than discussions of blockchain in food supply chains often do.

### 6.3. IoT, Cloud Systems, Edge AI, and Data Interoperability

The Internet of Things (IoT), meaning networked sensors and actuators capable of transmitting data without direct human operation, cloud-based data infrastructure, and edge AI, discussed in Section 5.3 as a latency-driven engineering choice, together form the connective infrastructure a smart egg processing system depends on regardless of whether it eventually incorporates a full digital twin or a blockchain traceability layer. The clearest demonstrated egg-relevant example of this infrastructure operating together remains Wu et al.’s henhouse monitoring system, which combined edge inference (Jetson AGX Orin), wireless data transmission (measured network latency and signal strength reported directly), and cloud storage and dashboarding (built on commercial cloud infrastructure) into one operating system, validated in the field across 60 commercial cages [25]. That this is, again, a farm stage rather than processing plant stage example is a limitation: we did not identify a processing line-specific study reporting comparable end-to-end IoT infrastructure metrics, which means the specific latency, throughput, and reliability figures a processing-plant deployment would need remain, at present, extrapolated from an adjacent application rather than directly measured for egg processing itself.

Data interoperability, the ability of sensors, control systems, and software from different vendors and different processing steps to exchange data in a common format, is a less visible but practically consequential obstacle to the integrated architecture we have described throughout. None of the egg-specific studies synthesized in this manuscript report testing interoperability across multiple sensor vendors, multiple control-system protocols, or multiple facilities, a gap directly connected to the calibration transfer problem discussed in Section 5.3 and to the standardization challenges Section 10 addresses in more depth. Without standardized data formats and communication protocols connecting the sensing layer (Section 3 (Part I)), the prediction layer (Sections 4 and 5 (Part I) [1]), and the process control layer (Section 2 and Section 3), the individually well-demonstrated components reviewed across this manuscript remain separable technologies rather than an integrated system, which is precisely the gap the Introduction identified as its central concern and which the concluding sections of this manuscript return to directly. Established industrial standards offer tangible ways to address this barrier. OPC UA (IEC 62541) provides a vendor-neutral, secure information model and communication layer between sensors, controllers, and software [31]; MQTT (ISO/IEC 20922) is a lightweight publish–subscribe protocol widely used for edge-to-cloud telemetry [32]; and ISA-95 (IEC 62264) defines the functional hierarchy and data models that link process control to manufacturing operations and enterprise systems [33]. These standards are often combined, for example OPC UA information models transported over MQTT. None has been evaluated for egg-processing lines in the literature searched, so they are presented as candidate architectural solutions to vendor lock-in, not as demonstrated ones.

## 7. Food Safety and Risk Management

Food safety has run through nearly every section of this review rather than sitting in one place, and this section’s task is to draw those threads together into a direct answer to a risk-management question: where, specifically, do the smart processing technologies surveyed in this manuscript improve an egg processor’s or a regulator’s ability to detect, predict, and respond to a safety hazard, and where do they fall short of that claim.

### 7.1. Pathogen Detection Versus Indicator Detection

The distinction Section 2.4 (Part I) [1] introduced as a general principle has a direct, practical risk management consequence. Biosensors of the kind reviewed in Section 3.8 (Part I) [1], built around antibody, aptamer, bacteriophage, or DNA-probe recognition elements, are designed to bind *Salmonella* or another target pathogen directly, and are therefore, in principle, detecting the hazard itself [34]. Every other sensing technology reviewed in this manuscript, hyperspectral and multispectral imaging, NIR, Raman, and fluorescence spectroscopy, and electronic nose volatile sensing, detects a physical or chemical correlate of spoilage or contamination risk, not the pathogen or hazard directly. For a risk management decision, this distinction determines what a given sensor reading can and cannot support: an elevated e-nose volatile signature is evidence of spoilage-associated microbial activity in general, not confirmation of a specific pathogen’s presence or absence, and a normal-looking hyperspectral freshness signature does not rule out contamination by a pathogen that has not yet produced the metabolic byproducts the sensor was calibrated to detect. Section 3.8 (Part I) [1] already established that even the direct detection biosensor literature remains squarely laboratory proof-of-concept, validated against spiked samples with known bacterial concentrations rather than the lower, more variable contamination levels and complex protein-and-lipid matrix an actual production line presents [34]. The practical risk management conclusion is that, at the current evidence tier, none of the sensing technologies reviewed in this manuscript can substitute for confirmatory microbiological testing in a formal food safety decision; their demonstrated role is triage and process guidance (flagging batches for further testing, or informing which processing intensity Section 2 or Section 3’s technologies should apply), not final safety verification.

### 7.2. Contamination and Spoilage Monitoring

Where sensing technologies have a more clearly demonstrated risk management role is in spoilage monitoring, tracking the freshness trajectory and volatile compound accumulation reviewed extensively in Sections 3 and 5.1 (Part I) [1], which is mechanistically closer to what these sensors actually measure. The e-nose and spectroscopic evidence discussed throughout this manuscript supports using these technologies as an early warning layer, flagging batches whose freshness trajectory has deviated from the expected pattern for further inspection or accelerated processing, a role consistent with the “predictive and preventive” framing Section 5 discussed at the level of process control generally. This is a genuine and demonstrable contribution to contamination and spoilage risk management, distinct from, and more defensible than, a direct pathogen detection claim.

### 7.3. Hazard Prediction and Process Verification

Hazard prediction, in the sense of forecasting the likelihood a batch presents an elevated safety risk before confirmatory testing is complete, is where the AI and machine learning methods reviewed in Sections 4 and 5 (Part I) [1] intersect most directly with food safety risk management, and where the validation concerns documented in Section 4.4 (Part I) and Table 4 (Part I) [1] carry the most direct consequence. A model that misclassifies a genuinely contaminated batch as low risk, a false negative, is a materially different kind of error from a model that misclassifies a safe batch as elevated risk, a false positive that costs throughput but not consumer safety, and Section 4.4 (Part I) [1] already noted that none of the egg–AI studies synthesized here report the class balance-adjusted, per class metrics (sensitivity specifically for the contaminated class, rather than overall accuracy) that would allow this review to assess which kind of error a given model is more prone to. This is a specific, food safety-relevant extension of the general validation gap we have documented throughout, not a new concern: a hazard prediction model’s overall accuracy figure is a poor proxy for its suitability in a risk management context, where the cost of a false negative and a false positive are not symmetric, and reporting practice across the literature synthesized here does not currently allow that asymmetry to be evaluated.

Process verification, confirming that a processing step actually achieved its intended safety outcome, is where the real-time monitoring architecture discussed in Section 5 and the process parameter optimization discussed in Section 3 would, if connected, provide the strongest food safety contribution we have identified: a system capable of verifying, batch by batch and in real time, that a non-thermal treatment achieved its target microbial reduction, rather than relying on periodic destructive sampling to infer that a fixed process schedule is generally effective. Section 5.2 noted that this specific connection, real-time sensing feeding back into verified, adjusted process control, has not been demonstrated for egg processing; the food safety implication of that gap is that current smart-processing evidence supports improved detection and monitoring, but not yet a demonstrated, closed loop verification system that would justify reducing reliance on the periodic destructive testing regimes non-thermal processing validation still depends on.

### 7.4. Traceability and Rapid Decision-Making

Traceability’s food safety role, tracing a contaminated batch back through the supply chain to its source and forward to every product it reached, is well established as a goal independent of the specific digital technology used to achieve it, and Section 6.2 already evaluated the egg-specific evidence for blockchain-based traceability in detail, concluding that a theoretical design exists for organic certification compliance specifically [30], but that no operational egg processing traceability system, blockchain-based or otherwise, was identified in our literature search at an evidence tier beyond the conceptual. Rapid decision-making, the capacity to act on a safety signal quickly enough to prevent a contaminated batch from reaching distribution, depends directly on the real-time sensing and edge computing infrastructure discussed in Section 5.3, where we found genuine, if farm-stage rather than processing plant-stage, evidence that sub-100 millisecond sensing-to-decision latency is achievable with current hardware [25]. Whether that infrastructure, demonstrated for grading decisions, can be extended to genuine food safety decisions, which typically require a higher evidentiary bar before a batch can be cleared or rejected, is a question the evidence base does not yet answer, and stating that limitation directly is more useful to a reader than implying the grading-line evidence already covers the food-safety use case. A further reason for caution is that food-safety decisions require higher statistical confidence thresholds and redundant or orthogonal sensor checks before a batch is cleared, and these requirements inherently add processing time compared with physical defect sorting, so latencies reported for grading cannot be assumed to transfer.

### 7.5. Validation Requirements Specific to Food Safety Applications

Every validation concern Section 4.4 (Part I) [1] raised for quality prediction generally applies to food safety applications with added force, because the consequence of an undetected failure is materially more serious. A food safety-oriented sensing or AI system would need, at minimum, external validation across multiple facilities, flocks, and seasons of the kind Section 4.4 (Part I) and Table 4 (Part I) [1] established is absent across the egg–AI literature synthesized here; explicit reporting of sensitivity for the hazard class specifically, not overall accuracy, given the asymmetric cost of false negatives discussed in Section 7.3; and validation against real-world contamination levels and matrix complexity rather than the spiked, laboratory-prepared samples the biosensor literature reviewed in Section 3.8 (Part I) [1] currently relies on. None of these represent a criticism of the individual technologies reviewed throughout this manuscript, whose underlying sensing principles and mechanisms are, in most cases, scientifically sound; they represent the specific, concrete gap between the laboratory proof-of-concept evidence we have synthesized and the validation standard a food safety application would need to meet before regulatory or industrial adoption, a gap Section 9 and Section 10 examine in more direct, practical terms.

## 8. Sustainability and Resource Efficiency

### 8.1. What Smart Processing Could Plausibly Contribute

The theoretical case for smart egg processing improving sustainability outcomes follows directly from mechanisms already established throughout this review rather than requiring new argumentation: non-destructive sensing (Section 3 (Part I) [1]) could reduce the destructive sampling this industry currently relies on for quality assurance; AI-based prediction (Sections 4 and 5 (Part I) [1]) could reduce over-processing by allowing a batch’s actual condition, rather than a worst-case assumption, to determine processing intensity; the AI-optimized non-thermal processing demonstrated in Section 3 could reduce energy use relative to fixed, conservatively over-specified thermal schedules; and improved freshness and spoilage monitoring (Sections 3 and 5.1 (Part I) [1]) could reduce food waste by extending accurately communicated shelf life and by flagging batches for earlier use rather than later, unplanned rejection. None of them has been demonstrated, on the evidence synthesized here, as a quantified, egg-specific sustainability outcome.

### 8.2. Demonstrated Versus Plausible: What the Evidence Actually Shows

The clearest concrete example we can point to is, again, the optimized ultrasound–thermal co-sterilization system of Fan et al. [17]. It achieved its target sterilization outcome at a peak temperature of 55 °C, against 64.5 °C for the conventional pasteurization comparison [17]. A lower peak processing temperature does not by itself demonstrate reduced energy consumption; at most it indicates a potential, plausible energy saving benefit, which remains an inference in the absence of measured energy data. The study reports no measured energy consumption, life cycle assessment, or carbon footprint figure. A lower temperature does not automatically imply lower energy use: total energy depends on holding time (11.5 min in the optimized ultrasound–mild-heat treatment versus 3 min for the conventional comparison), the electrical energy and conversion efficiency of the ultrasonic transducers, heat recovery efficiency, batch versus continuous operation, and throughput. Until measured energy or energy intensity data are reported, the benefit should be described as a potential or plausible energy saving effect, not a demonstrated reduction. We are not aware of any egg-specific study that reports such figures for a non-thermal or AI-optimized process compared with conventional thermal pasteurization.

Beyond this one study, our search for egg processing-specific sustainability evidence, energy consumption, water use, carbon footprint, or formal life cycle assessment comparing smart or non-thermal egg processing against conventional methods, did not identify a dedicated body of literature comparable in depth to what exists for sensing (Section 3 (Part I) [1]), AI prediction (Sections 4 and 5 (Part I) [1]), or even non-thermal processing efficacy itself (Section 2). This is a specific research gap; sustainability framing is considerably more developed for general (non-egg) non-thermal food processing: a review focused specifically on non-thermal technologies’ contribution to food-processing sustainability broadly identifies conventional thermal processes (pasteurization, sterilization, evaporation, refrigeration, freezing, and drying) as the most energy intensive category of technologies in the food industry generally, and frames non-thermal alternatives’ primary sustainability contribution as operating through three mechanisms: reduced energy consumption relative to heating and cooling-intensive thermal methods, reduced water consumption relative to processes that rely on water for heating or cooling, and reduced solid-waste generation [35]. That review draws on food processing broadly, not eggs, and is cited as context only.

### 8.3. What Would Be Required Before Strong Sustainability Claims Are Defensible

Three analyses would be needed before the plausible mechanism language of Section 8.1 could become a demonstrated-benefit claim. A formal life cycle assessment, accounting for energy and water inputs, waste outputs, and shelf life-extension effects across the full processing chain rather than a single unit operation, would be needed to determine whether a non-thermal or AI-optimized process’s plausible per batch energy advantage survives once equipment manufacturing, sensor infrastructure, and any additional computational load (the edge and cloud computing infrastructure discussed in Section 5.3 is not itself energy-free: continuous high frame rate computer vision, such as the 91.7 frames per second reported for edge inference by Wu et al. [25], and real-time cloud analytics draw non-trivial electrical power that must be set against any thermal energy savings in a net-zero processing line calculation) are accounted for. A techno-economic analysis, connecting the equipment, sensor, and AI infrastructure costs discussed in Section 9 to any demonstrated resource efficiency gain, would show whether a sustainability benefit is also economically viable at commercial scale. A carbon footprint assessment specific to egg processing would also be needed, because its processing chain (shell egg grading and washing, liquid egg pasteurization, drying, and packaging) differs from the fruit, vegetable, and dairy systems that dominate the existing non-thermal sustainability literature. Until these analyses exist, the sustainability contribution of smart egg processing should be described as plausible and mechanistically well-motivated, not demonstrated; the absence of egg-specific LCA, techno-economic, and carbon footprint data is among the more consequential and readily addressable gaps (Section 11).

## 9. Industrial Scale-Up and Economic Feasibility

Every technology we have surveyed sits somewhere on a path from laboratory bench to commercial deployment, and the evidence hierarchy framework introduced in Section 1 has tracked where each one currently sits throughout this manuscript. This section asks what stands between the laboratory and pilot scale evidence synthesized in this two-part review and industrial adoption, treating cost, infrastructure, and operational barriers as distinct obstacles.

### 9.1. Capital Cost

Equipment cost is the most concretely quantifiable barrier we can document, at least for the non-thermal processing technologies discussed in Section 2. However, essentially all available capital cost figures come from the broader food industry and not from egg processing-specific installations. We did not identify a published egg-specific capital cost analysis for any of the sensing or non-thermal technologies surveyed. High-pressure processing equipment, the most capital-intensive of the non-thermal technologies discussed in Section 2.1, has been estimated at approximately USD 0.5 to 2.5 million per industrial-scale unit, depending on vessel capacity (reported in the range of 55 to 525 L) and automation level [36]. This general food industry figure sets the order of magnitude for HPP-based liquid egg pasteurization and is substantially larger than the capital cost of conventional thermal pasteurization equipment, one reason thermal pasteurization remains dominant. Sensor hardware costs are comparatively modest: RGB cameras (Section 3.1 (Part I) [1]) are commodity hardware, whereas research-grade hyperspectral systems (Section 3.2 (Part I) [1]) remain markedly more expensive, and no sufficiently specific cost figure was identified.

### 9.2. AI Infrastructure, Data Requirements, and Skilled Workforce

Beyond the sensor and processing equipment itself, a functioning smart egg processing system requires computational infrastructure of the kind discussed in Section 5.3 and Section 6.3, edge computing hardware for latency-sensitive inference, cloud infrastructure for aggregation and dashboarding, and the data transmission and storage capacity to support both. Wu et al.’s henhouse system [25] is the most detailed available example of the infrastructure involved (Jetson AGX Orin edge device, 35 ms network latency, 91.7 frames per second), but it is farm stage evidence, and no processing line infrastructure cost analysis was identified in our literature search. Every AI model discussed in Sections 4 and 5 (Part I [1]) also requires a labeled training dataset, and most egg–AI datasets are small, single site, and laboratory-generated (Section 4.4 (Part I) [1]); industrial deployment would require a substantially larger and more diverse data collection effort, with an unquantified cost. HPP systems are documented in the general food processing literature as requiring specialized operation and maintenance skills, a training cost on top of the capital cost in Section 9.1.

### 9.3. Processing Line Integration, Throughput, and Reliability

Integrating a new sensing or processing technology into an existing egg processing line is a distinct engineering challenge from validating that technology in a standalone laboratory or pilot setup, and the throughput mismatch between the two is a recurring, specific obstacle we can document concretely. Commercial egg-grading lines operate at speeds measured in eggs per second across multiple parallel lanes, a throughput regime the industrial computer vision system reviewed in Section 5.1 was explicitly designed and deployed to match [23], but which several of the hyperspectral imaging studies reviewed in Section 3.2 (Part I) [1], working with data cubes described as easily hundreds of megabytes per egg at research-grade spectral resolution, were not evaluated against directly. This is why multispectral imaging was characterized in Section 3.3 (Part I) [1] as the industrially oriented endpoint of hyperspectral imaging: wavelength reduction responds directly to line speed requirements. Reliability, meaning consistent operation across extended production runs rather than the shorter validation periods most studies reviewed in this manuscript report, is a related concern the evidence base cannot speak to directly, since none of the sensing or AI studies synthesized here report multi-month or multi-shift reliability data of the kind an industrial quality assurance function would require before removing or reducing conventional inspection alongside a new sensing system.

### 9.4. Cybersecurity and Interoperability

The IoT, edge, and cloud infrastructure discussed in Section 5 and Section 6 introduces a category of risk that conventional, non-networked egg processing equipment does not carry: networked sensors, control systems, and cloud connections expand the attack surface available to a cybersecurity threat in ways that could, in principle, affect food safety critical process control decisions rather than merely data confidentiality. We did not identify a study, egg-specific or from the broader food processing literature surveyed, that evaluates cybersecurity risk for a smart egg processing or comparable food safety sensing system. This is a specific gap, not an implied absence of risk: networked sensing feeding a cloud dashboard and, in the still largely conceptual case of Section 5.2, an automated process control decision, is a system in which an unaddressed vulnerability could have food safety consequences beyond a data breach. Interoperability (Section 6.3) compounds this concern: a line assembled from several vendors’ systems without a common data and communication standard is harder to secure and to validate as an integrated system (see Section 10).

### 9.5. Regulatory Approval and Return on Investment

Regulatory approval pathways for non-thermal processing technologies remain, even for the more mature technologies discussed in Section 2, notably less standardized than the pathway for conventional thermal pasteurization. In the United States, processors adopting non-thermal or novel thermal technologies for low-acid or acidified foods are directed toward working with a recognized food Processing Authority and filing a scheduled process with the FDA under the low-acid canned food regulatory framework (21 C.F.R. Parts 108, 113, and 114), a framework built around thermal process validation and effectively repurposed for novel technologies in the absence of a technology-specific regulatory pathway, with regulatory guidance for at least one non-thermal technology group explicitly describing the regulatory status as unsettled and the underlying science as still developing [37]. The Food Safety Modernization Act’s preventive controls framework (21 C.F.R. Part 117) provides a further, more general regulatory layer requiring that any processing technology, thermal or non-thermal, be validated to demonstrate it does not introduce a food safety risk inadequately addressed by other preventive controls [37]. For liquid, frozen, and dried egg products, pasteurization in the United States is additionally governed by USDA Food Safety and Inspection Service regulations under the Egg Products Inspection Act (9 C.F.R. Part 590) [18], so any alternative process requires acceptance within that framework. The equivalent European framework combines the hygiene rules of Regulation (EC) No 853/2004 for foods of animal origin, which include egg products [38], the microbiological criteria of Regulation (EC) No 2073/2005 [39], and, where a technology or process changes the food significantly, the Novel Food Regulation (EU) 2015/2283, under which novel food status is determined case by case and novel foods undergo an EFSA safety evaluation before authorization [40]. This regulatory picture bears directly on return on investment: capital cost (Section 9.1) and validation cost (working with a Processing Authority and generating process filing documentation) must be weighed against benefits (functional property retention, energy or water savings, extended shelf life) that remain largely unquantified for eggs (Section 8.2). The economic case for industrial adoption of the AI-optimized non-thermal technologies discussed in Section 2 and Section 3 is therefore, at present, more plausible than proven. Table 4 summarizes the barriers identified in Section 9 with the solution each would require.

## 10. Standardization, Validation, and Regulatory Challenges

### 10.1. The Standardization Problem, Restated in Its Specific Forms

Section 4.4 (Part I) and Table 4 (Part I) [1] already established, in general terms, that the egg sensing and egg–AI literature synthesized here lacks external validation across independent flocks, facilities, and seasons. This section examines why that gap exists. It identifies the specific sources of variability responsible, rather than treating “lack of standardization” as a single undifferentiated problem. Hen breed and strain measurably affect egg composition, a fact that the authentication literature reviewed in Section 5.6 (Part I) [1] relies on directly: breed is one of the variables that the NMR models of Bischof et al. were trained to distinguish from housing system [41]. The same fact complicates any freshness or quality model trained on eggs from a single breed or strain when it is applied to another. Geographic variation and feeding regime interact with breed in ways the same authentication literature documents: Bischof et al. explicitly identified feeding regime and breed variation, alongside a regulatory provision permitting temporarily indoor-housed free-range hens to be relabeled as barn-housed, as plausible contributors to their model’s sharp accuracy decline on real supermarket samples [41], a finding we already discussed in detail in Section 5.6 (Part I) [1] and returns to here specifically because it is the clearest documented instance, among the studies synthesized in this manuscript, of a named biological and regulatory variable degrading model performance in a measurable, quantified way rather than as a generic caveat. Storage condition and egg age interact with all of the sensing modalities reviewed in Section 3 (Part I) [1], since nearly every freshness- and spoilage-related sensing technology we have discussed is fundamentally measuring a storage-time-dependent biochemical trajectory (Section 2.2 (Part I) [1]), which means a model’s applicability is bounded by the specific storage conditions, temperature, humidity, and duration, it was calibrated under, a limitation Section 5.1 (Part I)’s [1] discussion of constant temperature laboratory storage versus variable real-world cold chain conditions already illustrated directly through Kim et al.’s cold chain-specific study [42].

### 10.2. Sensor Calibration and Instrument Differences

Even holding the egg population constant, instrument-level variability presents a further, distinct obstacle to standardization. Section 3.2 (Part I) [1] and Section 5.3 both noted that hyperspectral imaging calibration transfer between instruments and facilities remains a documented, unresolved challenge in the egg-specific literature [27], and the same concern applies, in principle, to every spectroscopic technology reviewed in Section 3 (Part I) [1]: a PLS-DA or PLS regression model built on spectra from one manufacturer’s NIR or Raman instrument encodes that instrument’s specific optical and detector characteristics alongside the underlying chemical signal it is meant to capture, and transferring the model to a different instrument, even of the same nominal specification, typically requires a calibration transfer procedure we did not find directly evaluated in the egg-specific literature synthesized across Sections 3–5 (Part I) [1]. This is a specific, solvable engineering and chemometric problem with established general solutions in the wider analytical chemistry literature, but its application to eggs specifically, verifying that a freshness or authentication model transfers reliably across instruments from different manufacturers, has not been demonstrated in the studies we were able to identify. Established calibration transfer algorithms that future egg studies should evaluate include piecewise direct standardization (PDS), which maps spectra from a secondary instrument onto those of a primary instrument using local windows of wavelengths, and slope/bias correction (SBC), which adjusts predictions with a linear correction; both require a small set of transfer samples measured on both instruments [43,44]. These are off-the-shelf chemometric tools, but their performance on egg matrices, whose composition varies with breed, diet, and storage, remains to be tested.

### 10.3. Algorithm Reproducibility and Why Model Transfer Between Facilities Is Difficult

Reproducibility, in the specific sense of an independent research group or facility being able to retrain or directly apply a published model and obtain comparable performance, depends on a level of methodological detail: exact preprocessing steps, hyperparameter settings, train-test split composition, and software versions. Section 4.4 (Part I) [1] already noted that this level of detail is reported inconsistently across the egg–AI literature synthesized here. Combining that reporting gap with the biological and instrumental variability discussed in Section 10.1 and Section 10.2 explains, mechanistically rather than merely by assertion, why model transfer between facilities is difficult. A model trained at one facility encodes three things at once, often without the original researchers being able to fully disentangle which is which: the true underlying biochemical or physical relationship the technology is meant to capture, the specific hen population, feed, and storage conditions that facility’s eggs happen to represent, and the calibration characteristics of that facility’s specific sensing instrument. A second facility applying the model encounters a different combination of all three factors simultaneously. The authentication study of Bischof et al. [41], discussed at length in Section 5.6 (Part I) [1], remains our clearest quantified illustration of how severely performance can degrade when all of these factors shift at once, under external validation against genuinely independent, real-world samples rather than a held-out partition of the same training population.

### 10.4. Regulatory Acceptance and Standard Operating Procedures

Regulatory acceptance of a sensing or AI-based quality or safety determination, as opposed to acceptance of a non-thermal processing technology’s safety more generally (discussed in Section 9.5), faces an additional evidentiary bar we have not yet addressed directly: a regulator or industry standard-setting body evaluating whether a non-destructive sensing technology can substitute for, or supplement, conventional destructive testing needs the kind of cross-facility, cross-flock, externally validated evidence that Section 4.4 (Part I), Table 4 (Part I) [1], and this section have all established is currently absent from the published egg-sensing and egg–AI literature. Developing standard operating procedures for a specific sensing technology, calibration schedules, acceptable performance thresholds, required revalidation intervals when a facility’s egg supply or equipment changes, is a necessary precondition for regulatory acceptance that itself depends on the multi-facility validation studies the evidence base does not yet contain. This is not a criticism specific to any single technology reviewed in this manuscript; it is a structural gap affecting the sensing, AI, and non-thermal processing literature as a whole, and it is, correspondingly, one of the more clearly actionable research priorities we identify, taken up directly in Section 12’s research agenda.

## 11. Knowledge Gaps and Current Limitations

This section draws together the specific gaps identified throughout this two-part review, Section 2 in Part I [1] and Section 2, Section 3, Section 4, Section 5, Section 6, Section 7, Section 8, Section 9 and Section 10 in this paper, into one structured account, rather than restating them as a generic call for “more research” (Table 5). Each gap is stated in the same form: the problem itself, its practical consequence, the specific research needed to close it, and what a solution would look like if that research were carried out.

## 12. Future Research Agenda

The gaps identified in Section 11 translate into a research agenda structured by timeframe. Throughout, the agenda is stated as specific studies or actions rather than as generic calls for further work.

### 12.1. Short-Term Priorities (1–3 Years)

Three actions in this window require no new sensing hardware or processing technology, only more rigorous use of what this two-part review (Sections 3–5 in Part I [1]; Section 2, Section 3 and Section 4 in this paper) has already established exists. First, egg–AI research groups should establish shared, standardized benchmark datasets, pooling eggs across at least three independent facilities, multiple hen breeds, and a full annual cycle of seasonal variation, explicitly designed to support the external validation Section 4.4 (Part I), Table 4 (Part I), and Section 5.6 (Part I) [1] identified as absent; the Bischof et al. supermarket validation design [41] is a directly replicable template for how such a benchmark could be structured for other prediction tasks beyond authentication. These datasets, with raw spectra or images, reference measurements, and metadata on breed, feed, storage, and instrument, should be deposited in open repositories such as Zenodo, Kaggle, or Hugging Face with persistent identifiers and open licenses, following the FAIR (Findable, Accessible, Interoperable, Reusable) principles [46], so that independent groups can benchmark models fairly. In the same window, standardized chemometric calibration transfer protocols, such as piecewise direct standardization, should be validated across instruments from different manufacturers on these benchmark datasets, because instrument-level calibration transfer is a recognized gap (Section 10.2) that data pooling alone does not resolve. Second, journals and reviewers in this specific application area should require, as a condition of publication, reporting of both internal cross-validation and, where feasible, an independent external test, alongside class balance-adjusted metrics (sensitivity and specificity by class, not only overall accuracy) for any classification task with a safety-relevant minority class, directly operationalizing the reporting standard Section 11’s table calls for. Third, because initial egg-specific multimodal fusion studies have already been reported (Section 3.10 (Part I) [1]; Table 5), the priority is independent external validation of these existing fusion models, including their cross-facility generalizability across flock populations and seasons on the shared benchmark datasets described above, together with testing whether multimodal approaches consistently outperform individual sensing technologies on the same dataset; any new fusion model, for example hyperspectral imaging combined with electronic nose volatile sensing, should likewise report its performance against each unimodal baseline.

### 12.2. Medium-Term Priorities (3–5 Years)

This window requires the pilot scale infrastructure investment Section 9 discussed, and should prioritize connecting components we found validated only in isolation. The clearest specific target is a pilot scale demonstration coupling an inline sensor of the kind reviewed in Section 3 (Part I) [1] directly to a controllable non-thermal processing unit of the kind reviewed in Section 2, with an explicit, published decision rule connecting a measured or predicted attribute to a specific process parameter adjustment, extending Fan et al.’s offline optimization [17] into the real-time architecture Section 5.2 found does not yet exist. In parallel, the edge computing and cloud dashboard architecture Wu et al. demonstrated at the farm stage [25] should be replicated and evaluated specifically at the processing plant stage, reporting the same latency, throughput, and field reliability metrics for a processing line application, closing the farm-versus-plant evidence gap we flagged repeatedly in Section 5 and Section 6. A third priority is the first dedicated techno-economic and life cycle assessment for a specific smart egg processing technology combination, most plausibly built around the ultrasound–thermal system already validated at laboratory scale [17], since it is the one technology with an existing quantified safety and functional property result to build an economic and environmental analysis around.

### 12.3. Long-Term Priorities (5–10 Years)

The long-term agenda we can responsibly specify is bounded by what the medium-term work above would need to establish first, rather than described independently of it. Autonomous, adaptive processing, a system that adjusts non-thermal treatment parameters batch-by-batch based on real-time sensing without a human decision point, depends directly on the medium-term closed-loop pilot succeeding and being validated across the multi-facility, multi-season conditions Section 12.1’s benchmark datasets would need to represent. A fully integrated smart egg processing facility, combining multimodal sensing (Section 3 (Part I) [1]), fused and explainable AI prediction (Sections 4 and 5 (Part I) [1]), adaptive non-thermal processing (Section 2 and Section 3), real-time verification (Section 5), and digital traceability (Section 6) into one operating system, is the long-term endpoint the conceptual framework in Section 13 describes, and its feasibility rests on every gap identified in Section 11 being addressed rather than on any single breakthrough. Predictive food safety, forecasting contamination risk before it manifests rather than detecting it after the fact, depends specifically on closing the biosensor validation gap identified in Section 3.8 (Part I) [1] and Section 7.1 of this paper, since current biosensor evidence remains too far from real-world matrix and contamination level conditions to support a predictive claim. Digital certification and traceability at the scale envisioned by the blockchain proposal discussed in Section 6.2 [30] would require the interoperability standards that Section 11’s table identifies as absent to exist first, since a traceability system spanning farm, processor, distributor, and regulator cannot function without the data exchange standardization that we have identified as a prerequisite rather than a parallel workstream.

## 13. Smart Egg Processing 4.0: A Conceptual Framework

The evidence synthesized across this two-part review, Sections 2–5 in Part I [1] and Section 2, Section 3, Section 4, Section 5, Section 6, Section 7, Section 8, Section 9, Section 10, Section 11 and Section 12 in this paper, supports a conceptual framework connecting egg characterization, sensing, prediction, processing, and verification into a single pathway. We term it the Smart Egg Processing 4.0 framework. The framework is presented explicitly as a synthesis of what the evidence supports and does not support. It categorizes each stage by the evidence tier established throughout this manuscript, rather than functioning as an aspirational diagram that implies uniform readiness across every stage.

### 13.1. The Pipeline

The framework’s core pathway runs: raw egg characterization, establishing the incoming batch’s baseline external, internal, chemical, and safety attributes as defined in Section 2 (Part I) [1]; multimodal sensing, the combination of imaging and spectroscopic technologies reviewed in Section 3 (Part I) [1], deployed together rather than singly to resolve the spatial-versus-chemical trade-off by pairing the high spatial resolution of imaging with the high chemical specificity of spectroscopy, a trade-off Section 3.10 (Part I) [1] identified; data fusion, a step for which initial egg-specific implementations have been reported (Section 3.10 (Part I) [1]) but for which independent external validation and industrial scale application remain absent (Table 5, Section 11); AI/ML analytics, the classical and deep-learning methods reviewed in Section 4 (Part I) [1], applied with the validation discipline Section 4.4 (Part I) [1] established as necessary; quality and safety prediction, the task-specific outputs reviewed in Section 5 (Part I) [1], reported with the measurement-versus-prediction distinction Section 2.4 (Part I) [1] requires; process optimization, the AI-assisted parameter selection demonstrated for one technology combination in Section 3; non-thermal or hybrid processing, the technologies and their mechanisms reviewed in Section 2; real-time monitoring, the sense–analyze–predict–decide–act–verify loop Section 5 evaluated and found only partially demonstrated; functional and nutritional preservation, the outcome objective Section 4 established as co-equal with safety rather than secondary to it; verification, confirming a processing step achieved its intended outcome, discussed in Section 7.3; traceability, the digital record keeping layer evaluated critically in Section 6.2; and adaptive industrial processing, the long-term endpoint Section 12.3 described as dependent on every preceding gap being closed.

### 13.2. Cross-Cutting Dimensions

Three dimensions cut across every stage of the pipeline rather than belonging to any single one. Sustainability, evaluated throughout Section 8, depends on the energy, water, and waste implications of every processing and computational choice made at each stage of the pipeline. Our assessment, restated here, is that sustainability benefits remain plausible rather than demonstrated at essentially every stage. Food safety, evaluated throughout Section 7, is not a single pipeline stage but a standard that every stage must meet, from the measurement-versus-prediction rigor required at the sensing and prediction stages to the validated microbial inactivation evidence required at the processing stage. Data integrity and explainability, touched on in Section 4.3 (Part I) [1] and in Section 6.3 and Section 9.4 of this paper, span the sensing, AI, and traceability stages specifically, since a prediction, a process control decision, and a traceability record are each only as trustworthy as the data pipeline and model transparency underlying them.

### 13.3. Evidence-Tier Classification of the Framework’s Stages

Consistent with the evidence hierarchy we have applied throughout, each stage of the framework is classified here by what the synthesis actually supports, not by what the framework conceptually requires. Established technologies, meaning validated at least at pilot or industrial scale for egg applications specifically, include external computer vision-based grading and sorting (Section 5.1, [23]) and, more narrowly, point-based NIR spectroscopy’s core sensing principle, though not yet at the scale needed for full commercial calibration transfer (Section 10.2). Emerging technologies, meaning demonstrated at laboratory scale with reasonably consistent replication across multiple egg-specific studies, include hyperspectral and multispectral imaging for freshness and defect prediction (Sections 3.2 and 3.3 (Part I) [1]), Raman and fluorescence spectroscopy for freshness and authentication (Sections 3.5, 3.6 and 5.6 (Part I) [1]), electronic nose freshness prediction (Section 3.7 (Part I) [1]), and non-thermal processing technologies individually, HPP, PEF, ultrasound, cold plasma, and UV-C (Section 2), each validated for microbial efficacy at laboratory scale with technology-specific, documented limitations. Technologies requiring further validation before industrial consideration, meaning demonstrated in at most one or a small handful of egg-specific studies without independent replication, include egg-specific biosensors (Section 3.8, Part I [1]), egg-specific multimodal data fusion (Section 3.10 (Part I) [1]; Table 5), AI-optimized non-thermal processing (Section 3, resting on the single study by Fan et al. [17]), and edge computing real-time architecture at the processing plant stage specifically, as opposed to the farm stage evidence we found (Section 5.1). Future conceptual opportunities, meaning extensions of established building blocks that we discuss as plausible but that have not yet been demonstrated in any egg-specific study, include closed-loop process control connecting real-time sensing to automated process parameter adjustment (Section 5.2), digital twins for egg processing facilities (Section 6.1), and blockchain-based traceability beyond the theoretical design stage (Section 6.2).

**Figure 3:**
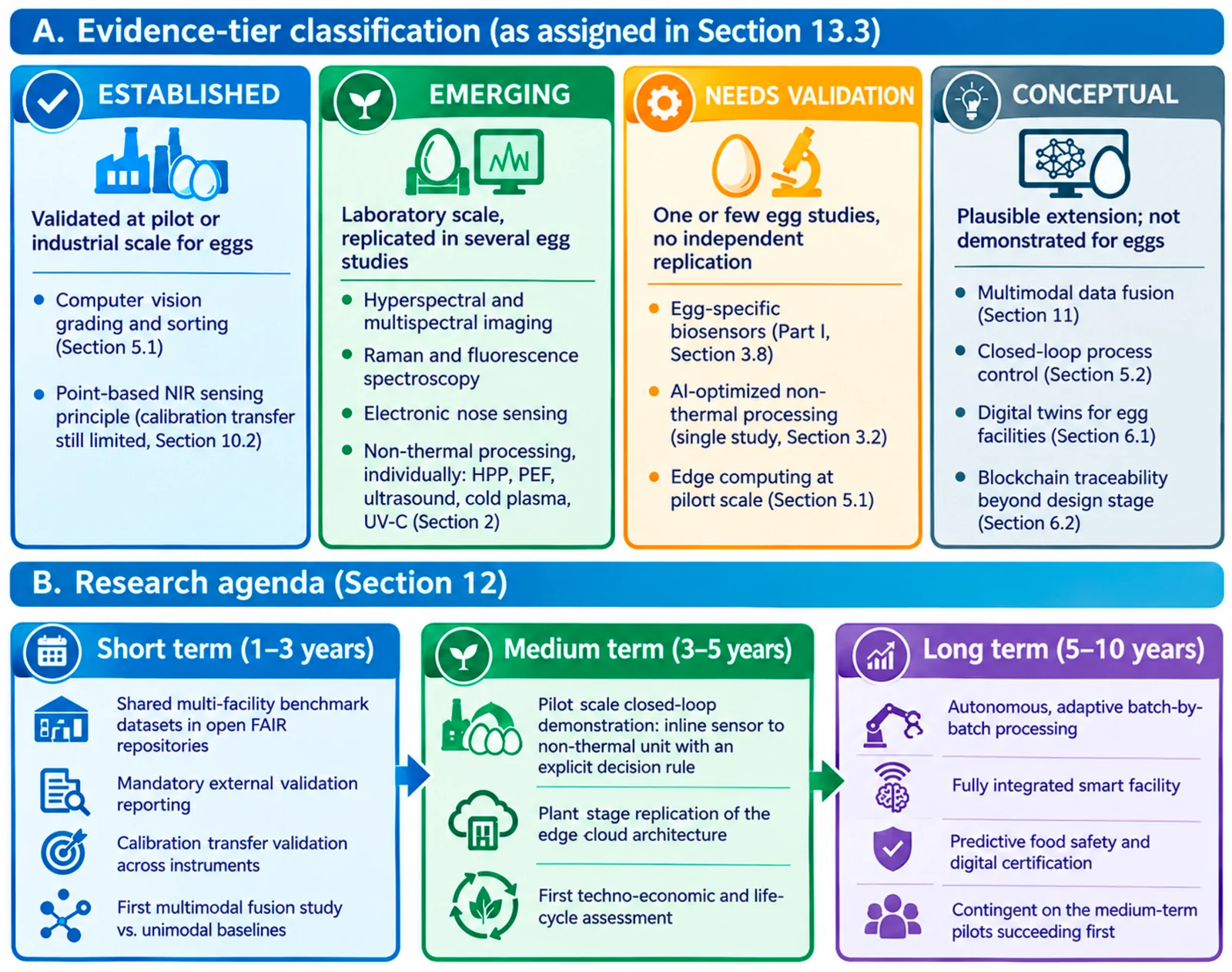
Technology readiness and research roadmap. Panel (**A**): four-tier evidence classification of the framework components exactly as assigned in Section 13.3 (established, emerging, needs validation, conceptual). Panel (**B**): short-, medium-, and long-term research agenda (Section 12). Original schematic prepared with Python 3.12.3 (Matplotlib 3.10.8).

This classification is, itself, the framework’s central claim. Smart Egg Processing 4.0 is not a single technology awaiting adoption, but an integration of components at markedly different evidence tiers. The most consequential research priority we identify, consistent with Section 11’s knowledge gap table and Section 12’s research agenda, is closing the distance between the tiers rather than further developing any single tier in isolation. Figure 3 consolidates this classification (Panel A) against the short-, medium-, and long-term research agenda set out in Section 12 (Panel B).

## 14. Conclusions

We set out to answer a specific question the Introduction posed directly. The question was not whether individual sensing, AI, and non-thermal processing technologies work for eggs, since Part I [1] and Section 2, Section 3 and Section 4 of this paper establish that most of them do, at least at laboratory scale and, in a smaller number of cases, at pilot or industrial scale. The question was whether the connective pathway linking sensing to prediction to process selection to functional verification has been demonstrated as an integrated system. On the evidence synthesized across this manuscript, it has not. This gap, not any single missing technology, is smart egg processing’s defining current limitation.

What we can state with confidence is considerable. Non-destructive sensing of egg quality and safety attributes is a mature and diverse field. It spans computer vision, hyperspectral and multispectral imaging, near-infrared, Raman, and fluorescence spectroscopy, and electronic nose volatile sensing, together with biosensors, which remain at an earlier stage of development. Each of these has an identifiable mechanistic basis (Part I) [1], and for several modalities there is genuine industrial deployment evidence (Section 5.1 of this paper). Machine learning and deep learning methods have been applied successfully across nearly every quality and safety prediction task we examined, freshness, defect detection, yolk characteristics, spoilage, and, most rigorously tested of all, authentication (Sections 4 and 5 (Part I) [1]). Non-thermal processing technologies, HPP, PEF, ultrasound, cold plasma, and UV-C, each offer a mechanistically distinct route to microbial inactivation with a correspondingly distinct, technology-specific trade-off against functional property retention, and the hurdle technology literature synthesized in Section 2.6 offers the most consistently supported general conclusion we can draw: in the laboratory studies reviewed, combining technologies at moderate intensity generally outperformed maximizing any single one. And at least one study, Fan et al.’s genetic algorithm-optimized ultrasound–thermal system [17], demonstrates that offline multi-objective parameter optimization of a non-thermal egg process—using response surface modelling combined with a genetic algorithm, and not real-time adaptive machine learning—treating safety and functional quality as simultaneous rather than sequential goals, is achievable and experimentally verifiable at laboratory scale, even if it remains a single, unscaled demonstration rather than an established practice.

What we cannot state with equal confidence is where this evidence base falls short of supporting the fully integrated system its own Introduction defined as smart egg processing. The validation gap documented in Section 4.4 (Part I) and Table 4 (Part I) [1] is not a peripheral methodological concern. It is illustrated most concretely by the authentication study of Bischof et al., where a model’s internal cross-validation ranking inverted entirely against real-world supermarket samples [41]. This gap is the central reason we have, throughout, distinguished laboratory proof-of-concept from pilot scale and industrial validation, rather than treating a high reported accuracy figure as evidence of readiness. The closed-loop connection between real-time sensing and automated process control, the specific capability that would move egg processing from end-product inspection to predictive and preventive control, has not been demonstrated for egg processing at any evidence tier beyond the conceptual in the literature searched (Section 5.2). And the economic and environmental case for adopting these technologies at industrial scale, addressed in Section 8 and Section 9, rests on plausible mechanism rather than on the life cycle assessment, techno-economic analysis, and multi-facility reliability data that would be needed to make it a demonstrated case rather than an inferred one.

These limitations define the research agenda rather than diminish the field’s progress. The most consequential near-term priority is closing the external validation gap: multi-facility benchmark datasets with externally validated performance metrics would transform the field’s ability to distinguish generalizable models from facility-specific ones (Section 12.1). The defining medium-term target is a pilot scale closed-loop demonstration connecting inline sensing to a controllable non-thermal processing unit—the specific empirical step that separates Smart Egg Processing 4.0 as a validated architecture from Smart Egg Processing 4.0 as a conceptual framework (Section 5.2 and Section 12.2). The framework developed in Section 13 is offered as an evidence-tiered map of which components are established, which are emerging, and which remain conceptual—so that investment can be concentrated on the connective steps found missing, not on further refinement of components already reasonably well understood in isolation.

Eggs occupy an unusual position in the food processing landscape. They are nutritionally dense, functionally versatile, and produced at a scale, approximately 100 million ton globally in 2024 [47], that makes even incremental improvements in processing efficiency, safety verification, or waste reduction consequential at an industry-wide level. The technologies we have surveyed offer a credible, evidence grounded path toward realizing that potential. Whether they do so depends less on any further breakthrough in sensing, algorithm design, or processing physics, all of which we found reasonably mature within their individual domains, than on the more difficult, less individually publishable work of connecting them: validating models across the facilities, flocks, and seasons real production actually spans, and building the integrated architecture that would let a measurement made in Section 3 (Part I) [1] genuinely inform a processing decision made in Section 2 or Section 3, in real time, at commercial scale. That is the specific, actionable gap we have identified, and closing it, rather than accumulating further isolated demonstrations of individual technologies, is this field’s most consequential next step.

## Figures and Tables

**Table 1:** Non-thermal processing technologies for eggs: mechanism, microbial effects, quality effects, advantages, and limitations.

Technology	Mechanism	Demonstrated Microbial Effect (Egg-Specific)	Key Quality/Functional Effect	Advantages	Principal Limitation
(**A**) **Liquid egg and egg-fraction processing (internal matrix)**					
High-pressure processing (HPP) (Section 2.1)	Membrane disruption and protein denaturation under 100–600 MPa	Reliable inactivation at 300–450 MPa, 0–50 °C, ≤30 min; full pasteurization requires intensities that risk coagulation [2]	Protein unfolding/aggregation scales with the same pressure driving inactivation	No bulk heating; genuine non-thermal mechanism	Antimicrobial efficacy and functionalnproperty risk are not separable at the same parameters
High-pressure homogenization (HPH)	Elevated pressure plus intense shear through a narrow valve	First-order inactivation kinetics vs. *S. Senftenberg* at 100 MPa (multi-pass) [4]	Reduced immunoreactivity via aggregation at 150 MPa; foaming capacity preserved [4]	Demonstrated preservation of a commercially relevant functional property despite structural change	Distinct equipment/process from standard HPP; narrower egg-specific evidence base
Pulsed electric fields (PEF) (Section 2.2)	Electroporation of microbial membranes under short high-voltage pulses	Not effective at optimized conditions in whole liquid egg due to viscosity [5]; effective in egg white and yolk fractions under other conditions [6]	Partial ovalbumin unfolding; altered sulfhydryl reactivity; enhanced antioxidant activity reported in derived peptides [6]	Potentially useful in lower-viscosity egg fractions; no bulk heating	Whole liquid egg’s viscosity is a specific, documented obstacle to effectiveness
Ultrasound (Section 2.3)	Acoustic cavitation generating localized shear, heat, and free radicals	Effective at 40 W/55 °C/5 min against *S. enteritidis* in liquid whole egg; additive (not synergistic) with HPP [5]	Foaming property first improves then declines with increasing time/temperature [17]	Effective as part of a hurdle approach; core mechanism for the one validated AI-optimized egg process [17]	Effectiveness depends on moderate heating; not a purely athermal technology in practice
(**B**) **Eggshell decontamination (surface matrix; UV-C is also applied to liquid egg in thin-film reactors)**					
Cold plasma (Section 2.4)	Reactive oxygen/nitrogen species and UV photons from ionized gas at ambient temperature	1 to >7 log CFU/egg reduction depending on configuration [9,10]	Cuticle integrity preserved in at least one configuration; bacterial physiological state affects susceptibility [10]	Ambient temperature surface decontamination; wide reported efficacy range reflects tunability	Surface/shell treatment only; not applicable to liquid egg internally; efficacy highly parameter-dependent
UV-C/pulsed light (Section 2.5)	Direct photochemical DNA damage	Demonstrated pathogen reduction on shell [11,12] and in liquid egg via engineered reactors [15]	Yolk carotenoid and fatty acid composition measurably shifted in at least one study [13]	Established mechanism; usable as a pasteurization pre-treatment to reduce subsequent thermal intensity [16]	Optical penetration depth is fundamentally limited in liquid, pigmented product

**Table 2:** Experimental conditions and outcomes of PEF studies on egg matrices, arranged to show the factors that may explain divergent results.

Study (Source)	Matrix	Target Organism	PEF Conditions	Temperature	Reported Outcome	Likely Explanatory Factor
Huang et al. [5]	Liquid whole egg	*Salmonella enteritidis*	30 pulses at 5.67 kV/mm (56.7 kV/cm)	55 °C	PEF not effective at the optimized condition; 3.2 log for the best HPP–ultrasound combination	High viscosity of whole egg, as attributed by the authors
Hermawan et al. [7]	Liquid whole egg	*Salmonella enteritidis*	25 kV/cm; 2.12 µs pulses; 200 pps; 250 µs total treatment time	Not specified for PEF step; heat step 55 °C, 3.5 min	Up to 1 log with PEF alone; 4.3 log with PEF plus heat	Little effect of PEF alone in whole egg; larger reduction when combined with mild heat
Studies summarized in Yogesh [6]	Egg white	*Salmonella enteritidis*	Nine exponentially decaying pulses at 35 kV/cm	Not stated in the review summary used here	3.5 log reduction	Albumen matrix and pulse waveform; lower viscosity than whole egg
Studies summarized in Yogesh [6]	Egg yolk	*Escherichia coli* O157:H7 and *Salmonella enteritidis*	PEF combined with elevated temperature	Elevated (value not stated in the review summary used here)	Synergistic inactivation	Heat–PEF synergy in yolk
Marco-Molés et al. [8] (quality study)	Liquid whole egg	Not applicable (quality only)	19, 32, and 37 kV/cm	Compared with heat pasteurization at 66 °C, 4.5 min	Foaming capacity retained at 19 and 32 kV/cm; reduced at 37 kV/cm and by heat	Field strength sets the functional quality ceiling

**Table 3:** Documented effects of thermal and non-thermal egg processing on nutritional and functional characteristics.

Technology	Protein Structure/Digestibility	Bioactive/Antioxidant Effect	Lipid/Carotenoid Effect	Functional Property (Foaming/Gelling/Emulsifying)	Evidence Tier
Conventional thermal pasteurization	Coagulation risk scales with time/temperature near the functional threshold [2]	Not a primary focus of the reviewed literature	Not reported as a primary focus	Foaming/viscosity change is the documented cost of adequate microbial safety [2]	Established (safety); functional cost well-documented
HPP/HPH	Structural unfolding and aggregation at pressures overlapping with inactivation effective range; reduced immunoreactivity via epitope masking [4]	Not directly reported in the egg-specific studies reviewed	Not reported	Foaming capacity preserved despite structural change at 150 MPa [4]	Laboratory
PEF	Partial ovalbumin unfolding; altered sulfhydryl group reactivity [6]	Enhanced antioxidant activity reported in resulting peptides in at least one line of evidence [6]	Not reported	Foaming capacity retained in whole egg at 19–32 kV/cm and reduced at 37 kV/cm [8]; gelling and emulsifying not reported	Laboratory
Ultrasound–thermal (optimized)	Not directly reported at the structural level	Not directly reported	Not reported	Optimum foaming window identified (~55–60 °C); superior to conventional pasteurization comparison at validated optimum [17]	Laboratory, experimentally validated
UV-C (shell and liquid)	Not reported	Not reported	Yolk carotenoid content and fatty acid composition measurably shifted alongside pathogen reduction [13]	Functional/nutritional composition evaluated directly for liquid-egg UV-C reactors [16]	Laboratory
Cold plasma	Not reported	Not reported	Not reported for eggs specifically in the literature reviewed	Not reported	Laboratory (safety only; functional property data not identified)
Non-thermal pre-treatment for bioactive peptides (general food evidence, not egg-specific)	Improved protease accessibility via conformational change [20]	Improved peptide yield and bioactivity reported across protein sources generally [20]	Not applicable	Not applicable	Evidence from other food systems; egg-specific confirmation not yet available (Section 11)

Note: “Not reported” indicates that the property was not reported in the cited sources; it should not be read as evidence that the property was tested and unaffected. Gelling and emulsifying capacity were not reported for most of the technologies listed. HPH, high-pressure homogenization; HPP, high-pressure processing; PEF, pulsed electric fields.

**Table 4:** Laboratory-to-industry barriers for smart egg processing and proposed solutions.

Barrier	Specific Problem	Proposed Solution
Capital cost	HPP equipment alone estimated at USD 0.5–2.5 million per unit [36], with no egg-specific capital cost analysis available for any technology reviewed	Egg industry-specific capital and operating cost studies; phased adoption starting with lower cost sensing (computer vision) before high-cost processing equipment (HPP)
AI infrastructure and data requirements	Most training datasets are small and single-site (Section 4.4 (Part I) [1]); edge/cloud infrastructure costs undocumented at processing plant scale	Multi-facility data sharing consortia (Section 12.1); processing plant-specific infrastructure cost and performance studies extending Wu et al.’s farm stage benchmark [25]
Skilled workforce	HPP and comparable non-thermal systems require specialized operation and maintenance capacity beyond conventional thermal line staffing	Technology-specific training programs developed alongside pilot scale deployments
Processing line integration and throughput	Hyperspectral data volumes and acquisition speed do not yet match commercial grading line throughput (Section 9.3)	Continued wavelength reduction (multispectral) approaches (Section 3.3 (Part I) [1]) purpose built for line speed constraints
Reliability	No multi-month or multi-shift reliability data identified for any sensing or AI system on an egg processing line	Extended field trials reporting performance over realistic industrial operating timeframes (Section 12.2)
Cybersecurity	Zero studies, egg-specific or general, evaluating cybersecurity risk for a smart food processing system (Section 9.4)	Dedicated risk assessment for networked sensing/control architectures before closed-loop systems (Section 5.2) are deployed at scale
Interoperability	No egg-specific study tests cross-vendor or cross-protocol interoperability (Section 6.3 and Section 9.4)	Adoption of common data exchange standards connecting sensing, prediction, and control layers
Regulatory approval	Non-thermal technologies rely on a thermal process validation framework repurposed for novel technologies, with unsettled regulatory status for at least one technology group [37]	Technology-specific regulatory pathways developed in coordination with the standardized validation datasets Section 12.1 calls for
Return on investment	Capital and validation costs must be weighed against largely unquantified benefits (Section 8.2)	Dedicated techno-economic analysis (Section 12.2), most plausibly built around the one technology combination with existing quantified results [17]

**Table 5:** Specific knowledge gaps identified across this review, their consequences, and the research needed to address them.

Gap	Problem	Consequence	Research Needed	Expected Solution
Small, single site datasets	Most egg–AI studies synthesized in Sections 3–5 (Part I) (Table 4 (Part I) [1]) calibrate on a few hundred to a few thousand eggs from one flock and one facility	Models may capture facility-specific or flock-specific artifacts rather than the generalizable biological signal	Multi-site data collection consortia pooling eggs across breeds, feeding regimes, and geographic origins into shared, sufficiently large datasets	Datasets large and diverse enough to support genuine external validation, not just larger internal train/test splits
Absence of independent external validation	Table 4 (Part I) [1] shows zero of the surveyed ML/DL studies test a trained model against eggs from a facility, flock, or season independent of the training data	Reported accuracy figures (Sections 4.4 and 5.6 (Part I) [1]) describe fit to the training population, not real-world performance; Bischof et al. [41] is the one study that tested this directly, and performance collapsed	Routine reporting of both internal cross-validation and independent external validation performance as a publication norm for egg–AI studies	A literature in which “accuracy” reliably predicts real-world performance, closing the gap Section 5.6 (Part I) [1] documented in detail
Lack of industrial scale (as opposed to laboratory or pilot) datasets	The evidence hierarchy (Section 1) classifies most non-thermal processing and AI-optimization studies (Section 3) as laboratory proof-of-concept	Laboratory validated safety and functional property outcomes may not hold at production line throughput and variability	Pilot and industrial scale replication of the laboratory findings synthesized in Section 2, Section 3 and Section 4, particularly Fan et al.’s ultrasound-thermal system [17]	At least one egg processing technology validated at each evidence tier from laboratory through industrial deployment
Limited real-time, closed-loop studies	Section 5.2 found no study coupling real-time sensing directly to an automated non-thermal process parameter adjustment	The “predictive and preventive” process control the Introduction identifies as smart processing’s defining feature remains conceptual, not demonstrated	A pilot study connecting an inline sensor (Section 3 (Part I) [1]) to a controllable non-thermal process (Section 2) with a documented decision rule	A demonstrated, even if narrow, instance of the full sense–analyze–predict–decide–act–verify loop for egg processing
Lack of standardized protocols and reporting	Section 10.3 found inconsistent reporting of preprocessing, validation splits, and hyperparameters across the egg–AI literature	Independent reproduction of published results, and fair comparison across studies, is frequently not possible	Field-wide adoption of a minimum reporting standard for egg-AI studies (dataset provenance, validation protocol, class balance metrics)	Egg–AI publications that can be directly compared and reproduced across research groups
Poor interoperability across sensing, AI, and control systems	Section 6.3 and Section 9.4 found no egg-specific study testing interoperability across vendors or protocols	The individually validated components reviewed throughout this manuscript remain separable technologies rather than an integrated system	Development or adoption of common data exchange standards connecting sensing, prediction, and process control layers	An integration layer allowing sensors, models, and controllers from different vendors to interoperate on one processing line
Limited multimodal sensing (data fusion)	Section 3.10 (Part I) [1] identified a strong conceptual case for combining spatial (imaging) and chemical (spectroscopic) modalities, and initial egg-specific multimodal fusion approaches have been reported in the literature (Section 3.10 (Part I) [1]); however, independent external validation of these approaches across multiple facilities and flock populations, and their industrial implementation, remain largely absent	Each sensing technology’s specific blind spot (Section 3.10 (Part I) [1]) goes uncompensated when deployed alone	Independent external validation of existing egg-specific multimodal fusion models across multiple independent facilities and flock populations, with performance benchmarked against each unimodal baseline	Externally validated multimodal fusion models demonstrating generalizable accuracy gains over unimodal sensing baselines across independent egg processing facilities
Limited AI explainability for egg-specific models	Section 4.3 (Part I) [1] found no egg-specific deep learning study applying SHAP, Grad-CAM, or comparable post hoc explanation methods	Plant operators have no documented basis for trusting that a deep model’s prediction is driven by the actual quality or safety signal rather than a confound	Routine application of the explainability methods reviewed in general food–AI literature [45] to egg-specific deep learning models	Egg–AI models whose predictions can be traced to a specific, verifiable image region or spectral feature
Limited economic analysis	Section 9.5 found no published techno-economic analysis specific to smart egg processing	The capital and infrastructure costs documented in Section 9.1 and Section 9.2 cannot yet be weighed against a quantified return, leaving adoption decisions without a clear evidence base	Techno-economic analyses connecting the equipment and infrastructure costs of Section 9 to the (currently largely unquantified) functional, safety, and throughput benefits documented elsewhere in this review	A defensible cost-benefit case, or the identification of conditions under which one exists, for specific technology combinations
Limited environmental assessment	Section 8.2 and Section 8.3 found no egg-specific life cycle assessment or carbon footprint study	Sustainability claims for smart or non-thermal egg processing rest on plausible mechanism rather than measurement	Formal life cycle assessment of at least one non-thermal or AI-optimized egg process against conventional thermal pasteurization	A quantified, egg-specific sustainability comparison rather than an inferred one
Limited long-term reliability data	Section 9.3 found no multi-month or multi-shift reliability study for any sensing or AI system deployed on an egg processing line	Whether current systems maintain accuracy over extended production runs, rather than the shorter validation periods typically reported, is unknown	Extended field trials reporting performance metrics over weeks to months of continuous industrial operation	Documented evidence of sustained accuracy (or a characterized degradation curve) over realistic operating timeframes
Limited integration of processing and sensing	Section 3.4 and Section 5.2 identified this as the central, defining gap we set out to address	Sensing, prediction, and processing remain three separately validated literatures rather than one connected system, which is precisely the gap motivating this review	Studies explicitly designed to connect Sections 3–5 (Part I)’s [1] sensing and prediction outputs to Section 2 and Section 3’s process parameter selection, batch by batch	The integrated architecture Section 13 describes conceptually, demonstrated for at least one technology combination
Unvalidated instrument-level calibration transfer	Spectroscopic models encode instrument-specific characteristics; transfer procedures such as PDS and SBC have not been evaluated for eggs (Section 10.2)	Models may fail when moved between instruments or facilities, even of the same specification	Evaluate PDS, SBC, and related algorithms on multi-instrument egg datasets (Section 12.1)	A validated calibration transfer protocol for egg spectroscopy [43,44]
Limited evidence connecting processing conditions to functional bioactivity	Section 4.2 found a mechanistic case, but no dedicated egg-specific study, for non-thermal pre-treatment enhancing bioactive peptide yield	Whether non-thermal processing can be deliberately tuned to enhance, rather than merely preserve, egg-derived bioactive compounds remains unanswered for eggs specifically	Studies applying the non-thermal pre-treatment approaches documented for other proteins [20] to egg protein hydrolysis specifically, measuring peptide yield and bioactivity directly	Quantified evidence of whether, and by how much, non-thermal pre-treatment improves egg-derived bioactive peptide yield over conventional hydrolysis alone

## Data Availability

No new data were created or analyzed in this study. Data sharing is not applicable to this article.
